# Extracellular Vesicles and Resistance to Anticancer Drugs: A Tumor Skeleton Key for Unhinging Chemotherapies

**DOI:** 10.3389/fonc.2022.933675

**Published:** 2022-06-23

**Authors:** Simona Pompili, Antonella Vetuschi, Roberta Sferra, Alfredo Cappariello

**Affiliations:** Department of Biotechnological and Applied Clinical Sciences, University of L’Aquila, L’Aquila, Italy

**Keywords:** chemoresistance, extracellular vesicles, metastasis, tumor recurrence, tumor microenvironment

## Abstract

Although surgical procedures and clinical care allow reaching high success in fighting most tumors, cancer is still a formidable foe. Recurrence and metastatization dampen the patients’ overall survival after the first diagnosis; nevertheless, the large knowledge of the molecular bases drives these aspects. Chemoresistance is tightly linked to these features and is mainly responsible for the failure of cancer eradication, leaving patients without a crucial medical strategy. Many pathways have been elucidated to trigger insensitiveness to drugs, generally associated with the promotion of tumor growth, aggressiveness, and metastatisation. The main mechanisms reported are the expression of transporter proteins, the induction or mutations of oncogenes and transcription factors, the alteration in genomic or mitochondrial DNA, the triggering of autophagy or epithelial-to-mesenchymal transition, the acquisition of a stem phenotype, and the activation of tumor microenvironment cells. *Extracellular* v*esicles* (EVs) can directly transfer or epigenetically induce to a target cell the molecular machinery responsible for the acquisition of resistance to drugs. In this review, we resume the main body of knowledge supporting the crucial role of EVs in the context of chemoresistance, with a particular emphasis on the mechanisms related to some of the main drugs used to fight cancer.

## Introduction

**Figure 1:**
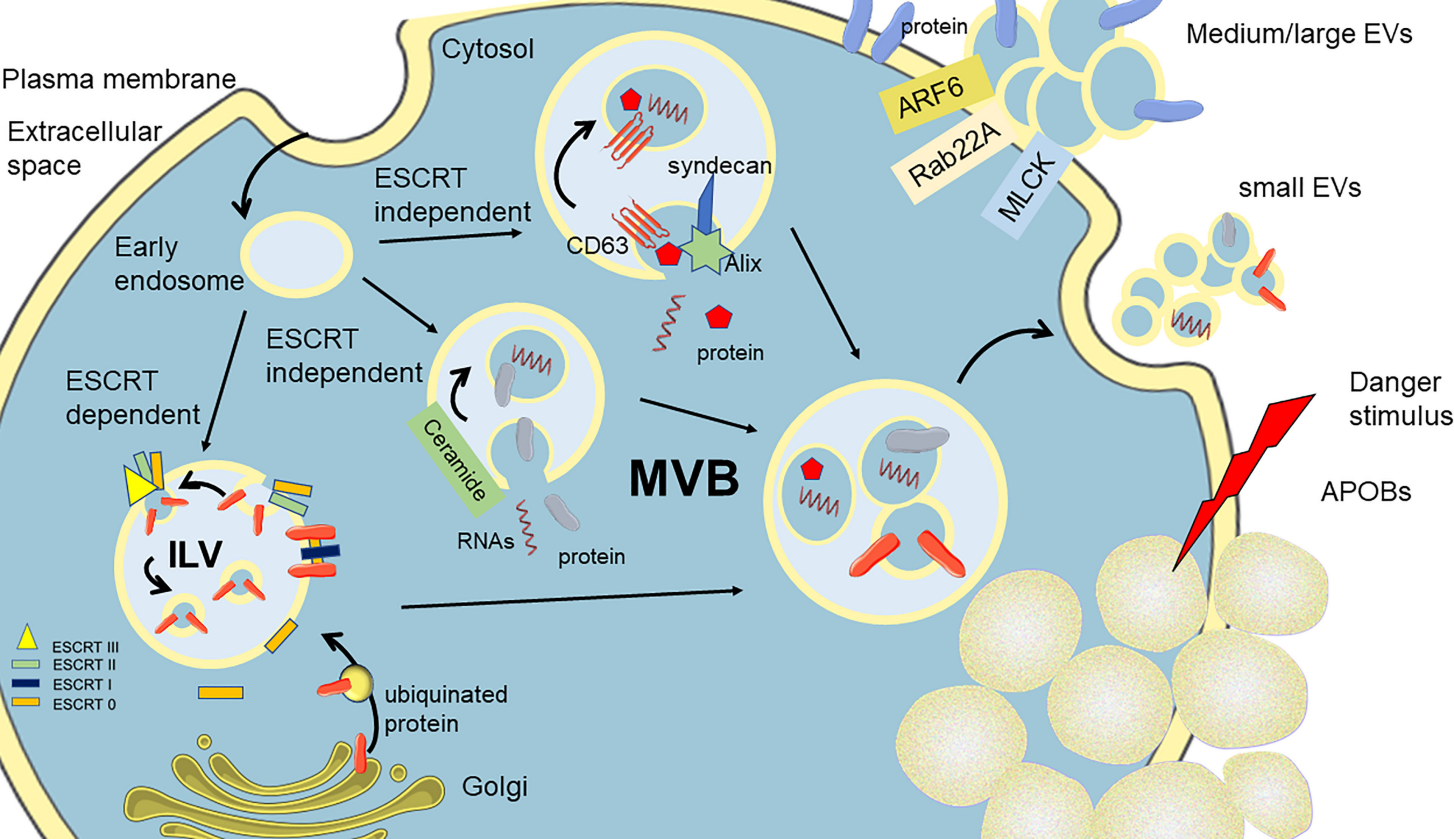
Schematization of the biogenesis of the three classes of EVs. Small EVs arise from the shedding of MVBs fusing with the plasma membrane (PM). Three main paths are described for the formation of MVBs, entrapping in the cytoplasmatic components of ILVs. Medium and large EVs are derived for the budding of PM, pulling the constituent of PM. Apoptotic bodies (APOBs) are released after an irreversible injury to the cell, triggering apoptotic cell death and the perturbation of PM. MVBs, multivesicular bodies; ILVs, intraluminal vesicles; ESCRT, endosomal sorting complexes required for transport.

Extracellular vesicles (EVs) are a heterogeneous population of double membrane-enclosed lipidic structures, which are actively secreted by eukaryotic and prokaryotic cells (1, 2). EVs are recently recognized as mediators of communication due to their molecular cargo consisting of biomolecules (lipids, nucleic acids, carbohydrates, and proteins) transferable to neighboring cells (3–5). EVs can be classified into three different subtypes according to their size, biophysical properties, and biogenesis: small EVs (exosomes), medium EVs (microvesicles), and apoptotic bodies (6) (**Figure 1**). Small EVs are nano-sized vesicles smaller than 150 nm, which originate from intraluminal vesicles (ILVs) through the formation of multivesicular bodies (MVBs) (7). As a next step, these ILV-containing MVBs can either be redirected to degradation in the lysosome or fused with the plasma membrane (PM), thus leading to the release of exosomes. The three main mechanisms of ILV formation are described. The first mechanism requires the presence of endosomal sorting complexes required for transport (ESCRT) complex members (8). These proteins have been described to select ubiquitinated proteins and segregate them into microdomains found on the endosomal membrane. ESCRT-0, ESCRT-I, and ESCRT-II are held responsible for the binding, through the tumor susceptibility gene 101 (TSG101), of specific cargoes selecting ubiquitinated proteins and segregating them into microdomains found on the endosomal membrane. Subsequently, these complexes recruit the apoptosis-linked gene 2–interacting protein X (ALIX), which aids in recruiting the ESCRT-III complex containing proteins involved in vesicle budding and the release from the plasma membrane. The second pathway, independent of ESCRT, requires only ALIX, and transmembrane proteins, such as syntenin and syndecan, which are responsible for recruiting tetraspanin CD63 (the main marker of small EVs) and other specific molecular cargoes (i.e., adhesion molecules, growth factors, and integrins) along with the interaction with proteins involved in the release from the cellular membrane (8). The third mechanism of ILV biogenesis mainly involves the participation of membrane lipid microdomains or lipid rafts. One of the main players is ceramide, generated by the neutral sphingomyelinase enzyme finally favoring the bending toward the lumen of the MVB membrane (9). Finally, MVBs can be degraded by fusion with lysosomes or can be shuttled to the membrane for the fusion and release of their cargo. On another side, medium/large EVs, also known as microvesicles, ectosomes, or microparticles, range between 50 and 1,000 nm. They are described to be released from the cell surface by blebbing from the plasma membrane. The biogenesis of EVs involves many partners, among which are small GTPases (such as ADP-ribosylation factor 6, ARF6), Ras-related proteins (Rab-22A), and phospholipases (PLD), the latter inducing a phospholipid redistribution and positioning of phosphatidylserines to the outer shell in EVs (10). The final recruitment of extracellular signal-regulated kinase (ERK) and the phosphorylation of the myosin light-chain kinase (MLCK) induce the invagination of the plasma membrane and EV release. Apoptotic bodies (APOBs) are the third subtype of EVs and vary in size, ranging from 50 to 2,000 nm in diameter, ultimately produced by the programmed cell death apoptosis (11). One of the main features of apoptotic bodies is that mechanisms for specific sorting of organelles, RNA, and DNA fragments can be detected, which are absent in other EV subtypes. APOBs are far to be inactive particles but are now shown to be lively involved in biological processes. During their biogenesis, EVs entrap different macromolecules, such as nucleic acids (DNA, mRNA, miRNAs, long non-coding RNAs), lipids, proteins (cytosolic factors, receptors, and ligands), and organelles, which are then shuttled to surroundings where they exert metabolic changes in target cells.

A physical/molecular interaction between EVs and cell membranes triggers the EV uptake. This interaction has been shown to occur *via* different routes, including a direct fusion between EVs and the plasma membrane (12), as well as EV internalization *via *clathrin-, lipid draft-, and caveolae-dependent endocytosis, macropinocytosis, and phagocytosis (13–15). Indeed, the EV uptake is likely dependent on many factors: the EV subtype, protein, and lipidic composition of the released EVs as well as the composition of the plasma membrane of recipient cells, cell metabolic status, and extracellular space conditions (i.e., pH, oxygen tension, and extracellular matrix components). The exchange of EVs is nowadays recognized as a crucial axis in the intercellular communication, exerting autocrine, paracrine, and systemic effects. EVs orchestrate physiological regulation in all tissues. The involvement of EVs is reported in many physiological processes such as angiogenesis [i.e., *via* the shedding of EV-encapsulated angiogenetic factors such as tetraspanin8, L-selectin, vascular endothelium growth factor receptor 1 (VEGFR1), and CD147], liver function and metabolism (i.e., asialoglycoprotein receptor-, apolipoprotein E/AV- and glutathione S-transferase-enriched EVs), bone resorption (i.e., pro-osteoclastogenic RANKL-positive EVs from osteoblasts), cornea wounding (i.e., fibronectin- and thrombospondin 1-enriched EVs from corneal epithelial cells), lung cell differentiation (i.e., EV-mediated shuttling and *de novo* transcription of pulmonary epithelial cell mRNAs), muscle regeneration (i.e., shuttling of α-Klotho transcript inducing muscle rejuvenating), bowel barrier integrity (epithelial cell-derived EVs alleviate gut injury after intestinal ischemia/reperfusion by miR-23a-3p), gut microbiota (*Escherichia coli* Nissle 1917 release vesicles positively modulates the intestinal epithelial barrier through upregulation zonulin-1/-2 and claudin-14), and immunity (macrophage-derived EVs contains alarmins orchestrating immune regulation) (16–24). Similarly, tissue dysfunctions and diseases are sustained by EV exchange including but not limited to stroke, obesity, skeletal muscle atrophy, colitis, and major depressive disorder (25–32). Finally, malignant transformation and cancer progression are fueled by a massive switch of EVs in many types of tumor (33–38). Carcinogenesis is a complex transformation of a cell by which specific traits or “hallmarks” are acquired, shifting from a healthy cell to a cancer one (39). According to Hanahan and Weinbergs’ theory, the mandatory hallmarks include the following: sustaining proliferative signaling, evading growth suppressors, resisting cell death, enabling replicative immortality, inducing angiogenesis, and activating invasion and metastasis. All of them are reported to be triggered by EVs. EVs from gastric cancer SGC7901 cells sustain proliferation by PI3K/Akt and MAPK/ERK activation; EVs are able to directly or epigenetically reduce the cytoplasmatic levels of phosphatase and tensin homologue (PTEN) to evade the growth suppressor (40–42). Many papers reported the mechanisms of resisting cell death induced by EVs: multiple myeloma cells reduced by EVs the levels of the pro-apoptotic protein Bcl-2-like protein 11 (Bim); the antiapoptotic protein survivin is shuttled in EVs from HeLa cervical carcinoma cells irradiated with a sublethal dose of proton, and both gastric- and bladder cancer-derived EVs suppressed the apoptosis of respective cancer or suppressive immune cells *via* the upregulation of Bcl-2 and cyclin-D1 expression and the downregulation of Bax and caspase-3 (43–48). The examples of replicative immortality triggered by EV cargoes are described for the shuttling of telomerase (TERT) transcript in target cells, as well as of TP53 and β-catenin (49–51). Angiogenesis is one of the most described event regulated by EVs, due to the enrichment in the EVs of VEGF, VEGFR, MMPs, and correlated agonists (52). Finally, metastatization support by EVs is widely investigated in a large body of literature reporting the plethora of their molecular cargo involved in the process, such as amphiregulin, C-X-C chemokine receptor type 4 (CXCR4), epidermal growth factor receptor (EGFR), interferon regulatory factor (IRF)-2, miR-105 (downregulating zonula occludens-1), and many others (53).

In this review, we will discuss the involvement of tumoral EVs in the insurgence of chemoresistance.

## Mechanism of Resistance to Chemotherapeutics

**Figure 2:**
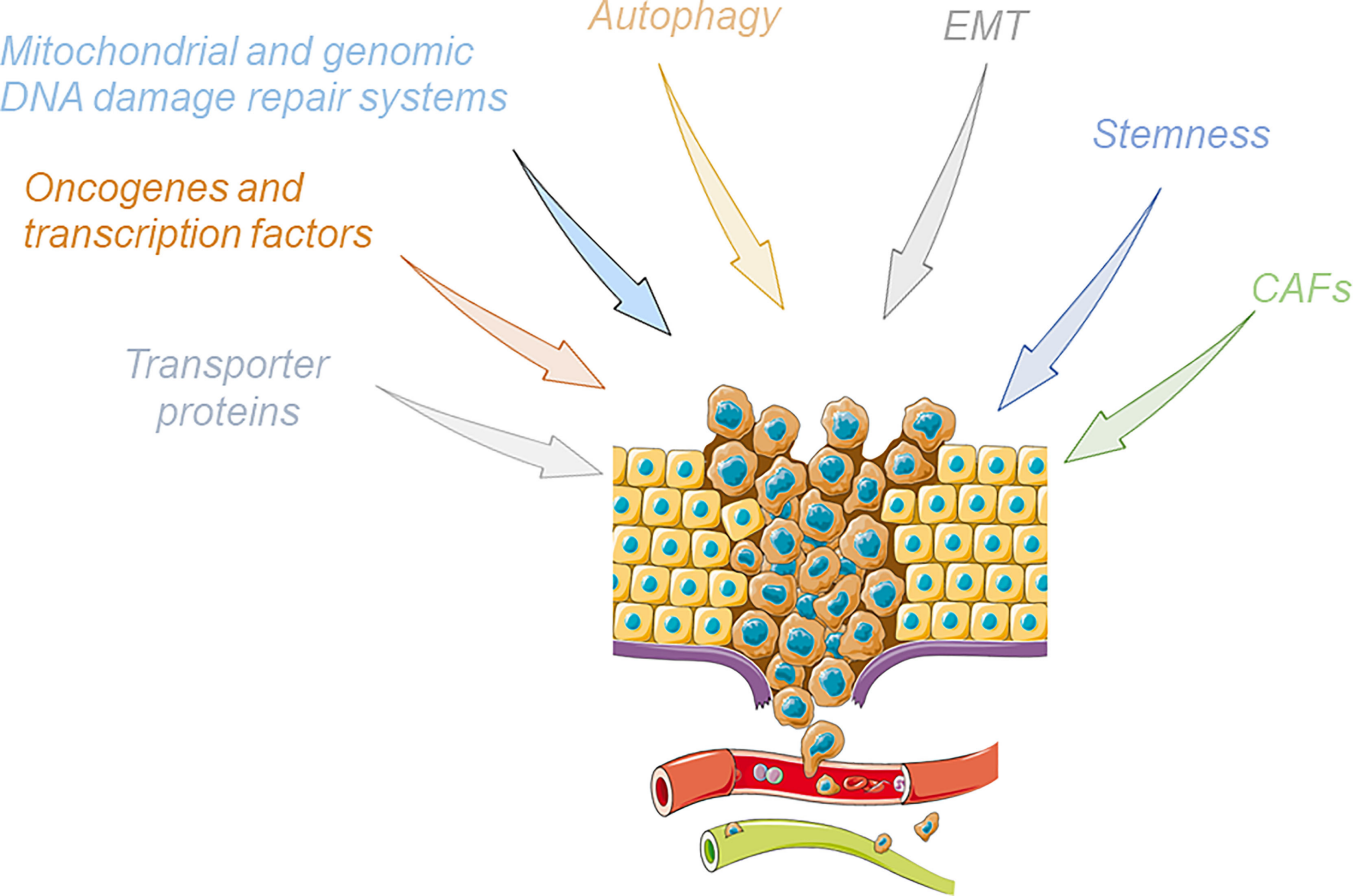
Cartoon summarizing the main mechanisms involved in the induction of the phenomenon of tumor drug resistance. Upon the influence of these pathways, drug-resistant cancer cells acquire an enhanced invasive ability, break away from the original tumor site, and finally metastasize to other organs through the blood or lymphatic systems. This figure was drawn with the support of the bioicons website (https://bioicons.com/).

Chemoresistance and radioresistance remain the main complications of cancer therapy, hindering the improvement of clinical outcomes for patients suffering from cancer since they cause cancer relapse and metastasis (54–56). Multiple mechanisms of resistance to drugs are reported, and the tight interconnection and support among them are one of the main issues for overcoming this crucial tumor feature. In the next section, we will provide an overview of the main mechanism of drug resistance reported in cancers (**Figure 2**).

### Transporter Proteins

The exchange across the plasma membrane is a pivotal mechanism of cellular homeostasis. The translocation of ions, lipids, amino acids, sugars, and xenobiotics occurs mainly through transporters or channels. The ATP-binding cassette (ABC) proteins and major vault protein (MVP) are the main players in these mechanisms. P-glycoprotein [P-gp, ABCB1, or multiple drug resistance 1(MDR1)] is an ATP-dependent efflux pump widely expressed in many tissues: capillary endothelial cells, intestinal epithelium, liver cells, and the renal proximal tubule (57). P-gp is one of the most powerful detoxification tools for cytotoxic drugs in cancer cells *via* direct efflux. P-gp overexpression has been observed in different kinds of hematological and solid tumors, such as leukemia, neuroblastomas, and ovarian and breast cancers, demonstrating its contribution to chemoresistance (58, 59). The ABCG2 encodes for another member of the ABC superfamily, also known as breast cancer resistance protein (BCRP) (60, 61). It has been reported that ABCG2 is an estrogen-inducible gene, associated with a higher tolerance of breast cancer cells against cytotoxic drugs (i.e., mitoxantrone) (62). Major vault protein (MVP) is a protein localized to a nuclear pore as a ribonucleoprotein with a hollow barrel-like structure responsible for gating ribosomes, hormones, and drugs (63). MVP was first discovered as a new 110 kD drug transporter in doxorubicin-resistant lung cancer cells, and it was later reported in many types of tumors (64). In triple-negative breast cancer cells MDA-MB-231, MVP has been demonstrated to be upregulated by the Notch1 intracellular domain and the activation of the AKT pathway and promoting the epithelial-to-mesenchymal transition (EMT) and the chemoresistance of cancer cells (65).

### Oncogenes and Transcription Factors

The overactivation and mutations of genes encoding for proteins involved in pivotal cellular processes (proliferation, survival, and transformation) is a frequent strategy of cancers to overcome the effect of cytotoxic drugs. The most upregulated pathways in chemoresistance are JAK/stat3, PI3K/Akt/mTOR, Src/FAK/ROS, and SOS/Grb2/Ras cascades. In turn, oncogenes can be upstream activated by receptors. For example, all the above pathways can be commonly activated by the EGFR. Accordingly, the overexpression or gain-of-function mutations of EGFR are reported in different types of aggressive and chemoresistant cancers. EGFR promotes metabolic processes critical for cancer cell proliferation both directly by phosphorylating rate‐limiting enzymes or indirectly through the activation of the MYC transcription factor and of the AKT signaling cascade (66–68). A mutated p53 is another common feature of many cancers (69). The protein p53 is involved in the sensitivity of cells to DNA-damaging drugs through DNA damage-response sensors ataxia telangiectasia mutated protein (ATM) and ataxia telangiectasia and Rad3-related protein (ATR) and their downstream cell cycle regulator checkpoint kinases 1 and 2 (Chk1 and Chk2) (70, 71). Some mutated p53 forms are very stable to degradation and ubiquitination and heterodimerize with wild-type p53, working as a dominant-negative able to disrupt most or all normal p53 functions, such as apoptosis or cell cycle arrest (72–74). Many mutated p53 forms can stimulate the mammalian target of rapamycin (mTOR) and block autophagy, leading to proliferative and anti-apoptotic responses in breast and pancreatic cancers (66). On other hand, the nuclear factor kappa-light-chain-enhancer of activated B cells (NF-κB) is another master player in dampening apoptosis induced by a variety of stimuli, including tumor necrosis factor-α (TNF-α), γ-radiation, and chemotherapeutics (75). Cancer usually expresses high levels of constitutive NF-κB activity and the exposition to cytotoxic agents increases NF-κB activity, resulting in cell growth and survival and finally resistance to the therapeutic agents. NF-κB induces the overexpression of downstream anti-apoptotic genes, such as the radiation-inducible immediate-early gene (IEX-1L), the inhibitor of apoptosis (IAP), and growth arrest and DNA damage-inducible 45 beta (Gadd45β), B-cell lymphoma-extra large (Bcl-xL), cyclin D1 and c-Myc, and many others, finally contributing to the chemoresistance. Thus, the NF-κB signaling pathway could be a potent target for improving the chemosensitivity of the tumor cells (76).

### Mitochondrial and Genomic DNA Damage Repair Systems

Many types of tumors harbor somatic mutations in the mitochondrial genome (mtDNA), resulting in mitochondrial dysfunction. Many mutations in the mitochondrial genes of cancer cells overload mitochondrial activity mainly because cancer cells shift their metabolism, requiring more glycolysis or oxidation. Proliferator-activated receptor gamma co-activator (PGC)-1α and mitochondrial transcription factor A (TFAM) are overexpressed in cisplatin-resistant ovarian cancer; similarly, PGC-1β confers the chemoresistance of lung cancer cells to cisplatin associated with mtDNA mutations (77, 78). Mitochondrial dynamics (fission and fusion) are critical for metabolic adaptations. Mitochondrial fusion with efficient ATP production was frequently observed in chemoresistant cancer cells (79). Dynamin-related protein 1 (DRP1) promotes mitochondrial fission, and its hyperexpression induces chemoresistance in lung, breast, thyroid, and colon cancers (80–84). DNA damage is the goal of many chemotherapeutic drugs, acting as alkylating (i.e., cisplatin) or antimetabolites (5-fluorouracil) to the DNA molecules. Cancer cells counteract their effects through a strengthening of the DNA repair, occurring mainly *via* nucleotide excision repair and base excision repair machinery. Excision Repair Cross-Complementation Group (ERCC) 1 is involved in the nucleotide excision repair pathway and has been reported to be associated with chemoresistance in melanoma and ovarian cancer and colorectal cancer (CRC) (85–87). Similarly, ERCC2 also supports chemoresistance in ovarian cancer (88). Reversionless 3-like (REV3L), the catalytic subunit of DNA polymerase ζ, modulates sensitiveness to 5-fluorouracil in lung and esophageal carcinoma (89, 90).

### Autophagy

Cells upon nutrient starvation, hypoxia, cellular stress, or metabolic alteration initiate autophagy to degrade cellular-damaged organelles and recycle amino acids or fatty acids *via* autophagosome formation. This adaptation strategy aims to favor cell survival and proliferation and is therefore adopted by cancer cells to fight drugs. CRC tissues were reported to be characterized by a significantly higher expression of autophagy-related genes such as Beclin-1, microtubule-associated protein 1A/1B-light chain 3 (LC3), and Rictor, which levels are positively correlated with the level of MDR-1 (91). The pro-survival role of autophagy was also confirmed in breast, ovarian, esophageal, lung, prostate, glioma, bladder, renal, and pancreatic cancers (92). The crucial role of autophagy was confirmed by the use of autophagy inhibitors, such as 3-methyladenine, able to sensibilize tumor cells to drugs (93). Human leukemia cells resist doxorubicin and vincristine by secreting high mobility group box 1 (HMGB1), responsible for overexpressing LC3-II in cancer cells (94). HMGB1 overexpression also contributed to the chemoresistance of neuroblastoma cells by inducing Beclin-1-mediated autophagy (95).

### Epithelial-to-Mesenchymal Transition

EMT is a complex process wherein epithelial cells depolarize, lose their cell–cell contacts, and acquire an elongated, fibroblast-like morphology. This mechanism is a means by which tumor cells increase their metastatic potential and can be triggered by extracellular signals (collagen, hyaluronic acid, and integrins), growth factors and cytokines (TGF-β, VEGF, EGF, and HGF), non-coding RNAs, or hypoxia (96). Under EMT, cancer cells enhance mobility, invasion, and resistance to apoptotic stimuli. Finally, through EMT, tumor cells acquire stemness (see next paragraph) and chemoresistance. Targeting EMT could indeed be an effective approach to obstacle chemoresistance (97). Colon cancer cells have been reported to encounter EMT and gain doxorubicin chemoresistance *via* the upregulation of TGF-β signaling (98). In hepatoma cells, gemcitabine supports EMT upon PDGF-D trigging, while oxaliplatin exposition induces EMT through BMP4/MEK1/ERK/ELK1 pathway activation (99, 100). In breast cancer cells MCF-7, EMT driven by Snail upregulation is reported to be associated with 5-fluorouracil insensitiveness (101).

### Stemness

The concept of stemness in the cancer field is nowadays widely accepted, and cancer stem cells (CSCs) are the actual dogma for the basis of cancer recurrence and chemoresistance (102). The definition of CSCs is the same as the normal tissue stem cells: the ability of a small subset of cells in a tissue having the capacity for self-renewal and to reform in a host the complete tissues containing all the cellular hierarchy from whence the stem cells were derived (103). Similarly to stem cells, CSCs can be purified as a poorly or negatively stained side population (SP) by flow cytometry, so-called because of their characteristic hallmark to exclude the Hoechst from the nucleus, while other tumor cells are highly positive for the nuclear DNA staining (104). This feature is associated with a high expression of the ATP-binding cassette transporter protein ABCG2/Bcrp1 (105). The other molecular signatures of CSCs reported included Oct4, Nanog, Sox2, ALDH, CD44, CD117, CD133, Notches members, and many others (106–109). CD44, a hyaluronic acid receptor, is highly expressed by cancer stem cells and interacts with the WNT/β-catenin pathway, leading to more aggressive tumors in pre-clinical models and patients suffering from CRC (110). CD44-expressing ovarian cancer stem cells are more resistant to platinum salts and to paclitaxel (PTX) than CD44-negative cells (111). CD133-expressing ovarian cancer stem cells have been shown to have increased engraftment capacities with chemoresistance to cisplatin (112).

### Cancer-Associated Fibroblasts

CAFs are vital constituents of the tumor microenvironment, a special stroma that interacts with cancer cells to promote tumorigenesis and progression. CAFs are recognized as potential targets for anti-cancer therapy since they are described to promote both cancer metastasis and chemotherapy resistance. Tumor cells depend upon the tumor stroma since it provides nutritional support and survival signals for tumor maintenance and proliferation. Upon certain stimuli, the fibroblast inside tumor stroma becomes “activated” (113). Accordingly, fibroblasts acquire different morphology and expression profiles (114). These CAFs produce growth factors that promote tumor growth, angiogenesis, and the recruitment of protumorigenic inflammatory cells. For example, CAFs specifically produce fibroblast activation protein alpha (FAP), changing different processes such as the extracellular matrix remodeling and composition as well as immune surveillance. CAFs can also affect the sensitivity of tumor cells to chemotherapy or radiotherapy (115, 116). The main mechanisms reported for CAF-mediated chemoresistance are the release of secreted factors, the promotion of cancer stemness, the modulation of cancer metabolism, and the induction of immune escape. The pleiotropic cytokine interleukin(IL)-6 is one of the main CAF-secreted factors. In esophageal squamous cell carcinoma (ESCC), IL-6 released by CAFs increased the chemoresistance of ESCC to cisplatin by increasing the chemokine receptor CXCR7 expression in tumor cells through the STAT3/NF-κB axis (117). Similarly, CAFs can release IL-8, promoting chemoresistance to cisplatin in human gastric cancer *via* NF-κB activation and ABCB1 upregulation (118). CAFs support the chemoresistance of tumor cells by promoting stemness. In colon cancer, Lotti *et al.* showed that CAFs upon the FOLFOX protocol released IL-17 which sustains the reservoir of CD44-positive self-renewing tumor-initiating cells (119). In breast cancer, CAFs secreted soluble factors such as activin A, insulin growth factor (IGF)-1, and leukemia inhibitory factor (LIF), all of which enhanced CSC proliferation and self-renewal *via* the activation of hedgehog signaling (120). Cancer and the tumor microenvironment acquire peculiar metabolic needs switching toward aerobic glycolysis (Warburg effect) (121). In lung carcinoma, EGFR- or MET-expressing cancer cells exhibited an elevated glycolysis activity and increased production of lactate that induced CAFs to secrete large amounts of HGF through an NF-κB-dependent mechanism. Subsequently, HGF activated MET-dependent signaling and enabled cancer cells to resist tyrosine kinase inhibitors (122). The escape from immune surveillance is a pivotal pro-survival event adopted by cancer cells, and CAFs can directly promote this phenomenon. In fact, in pancreatic cancers, CAFs have been reported to actively switch polarizing macrophages toward the immunosuppressive M2 phenotype by the release of IL-8, the granulocyte-macrophage colony-stimulating factor (GM-CSF), and monocyte chemoattractant protein-1 (MCP-1) (123). In breast cancer, CAFs over-express chitinase-3-like-1 (Chi3L1), a secreted glycoprotein, involved in macrophage recruitment and M2 polarization (124). In fact, genetic *in vivo* ablation of Chi3L1 in fibroblasts reduced tumor growth and macrophage recruitment while enhancing tumor infiltration by T cells.

## Chemoresistance and EVs

A consistent body of evidence showed that EVs are an invaluable tool for tumor cells for protecting against cytotoxic agents. Generally, EVs sequester and extrude far from the tumor cells a drug, gaining resistance to chemotherapy. Shedden *et al.* measured this feature by the correlation of a “vesicle shedding index” with the sensitivity of breast cancer cells MCF7 for a range of drugs (125). Accordingly, other authors reported that the release of EVs from resistant cells is higher compared to parental sensitive cells in different cancer cell lines, such as ovarian and pancreatic cancers (126, 127). The higher vesiculation allowed to export drugs, allowing the cells to be more resistant. Furthermore, the tumor EVs can be “upgraded” with specialized molecular machinery to more efficiently load drugs inside. Cancer cells, such as MCF7, overexpress upon doxorubicin exposition the *ABC* genes (encoding for ATP-binding cassette transporters known to confer resistance to multiple drugs) (128). The protein is found not only as a membrane transporter, to extrude drugs from the cytoplasm to the extracellular space, but is also present on the surface of EVs (129). Interestingly, the orientation of the protein on EVs can be reversed (130, 131). This feature allows importing the drugs inside EVs before their release from cells and improves the resistance of cancer cells to chemotherapeutics. Moreover, EVs can dampen the effectiveness of biological drugs. In fact, EVs act as a decoy or antagonist of monoclonal antibody-based therapies.

**Figure 3:**
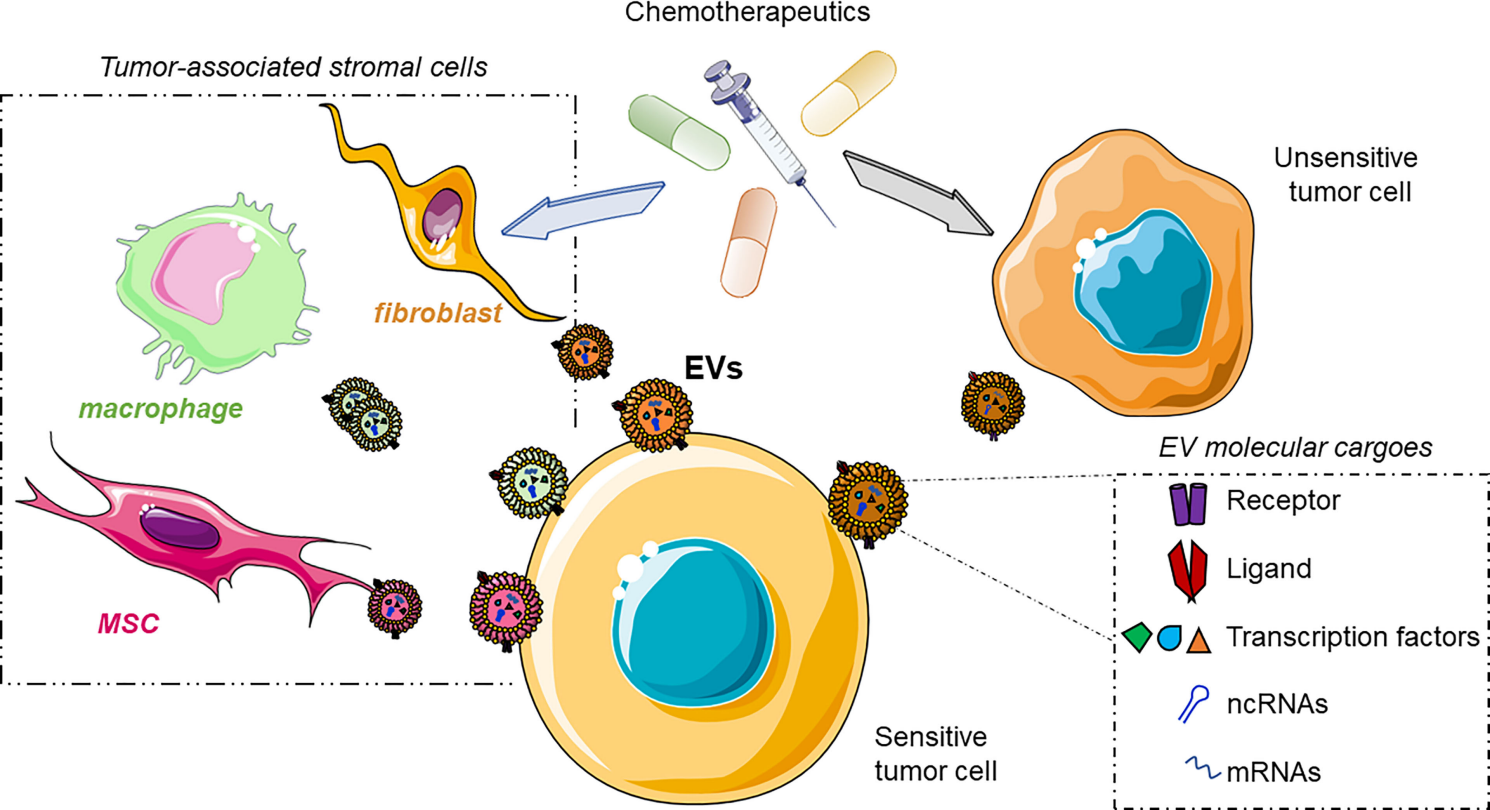
Cartoon illustrating the involvement of EVs in the spreading of chemoresistance among the cells of the tumor microenvironment (TME). The drugs induce the modification of the gene expression of primary tumor cells or TME cells. These cells release EVs enriched in the molecular players involved in the acquisition of drug resistance in the EV-donor cells, finally taken up by the sensitive tumor cells acquiring the insensitiveness toward the chemotherapeutic. Bottom-right box: molecular effectors involved in drug resistance shuttled through the EVs. ncRNAs, non-coding RNAs; mRNAs, messenger RNAs. This figure was drawn with the support of the bioicons website.

In the next section, we will discuss the EV-based strategy adopted by cancer cells to overcome chemotherapeutic agents (**Figure 3**).

## Mechanisms of Drug Resistance Activated by EV Molecular Cargoes

**Figure 4:**
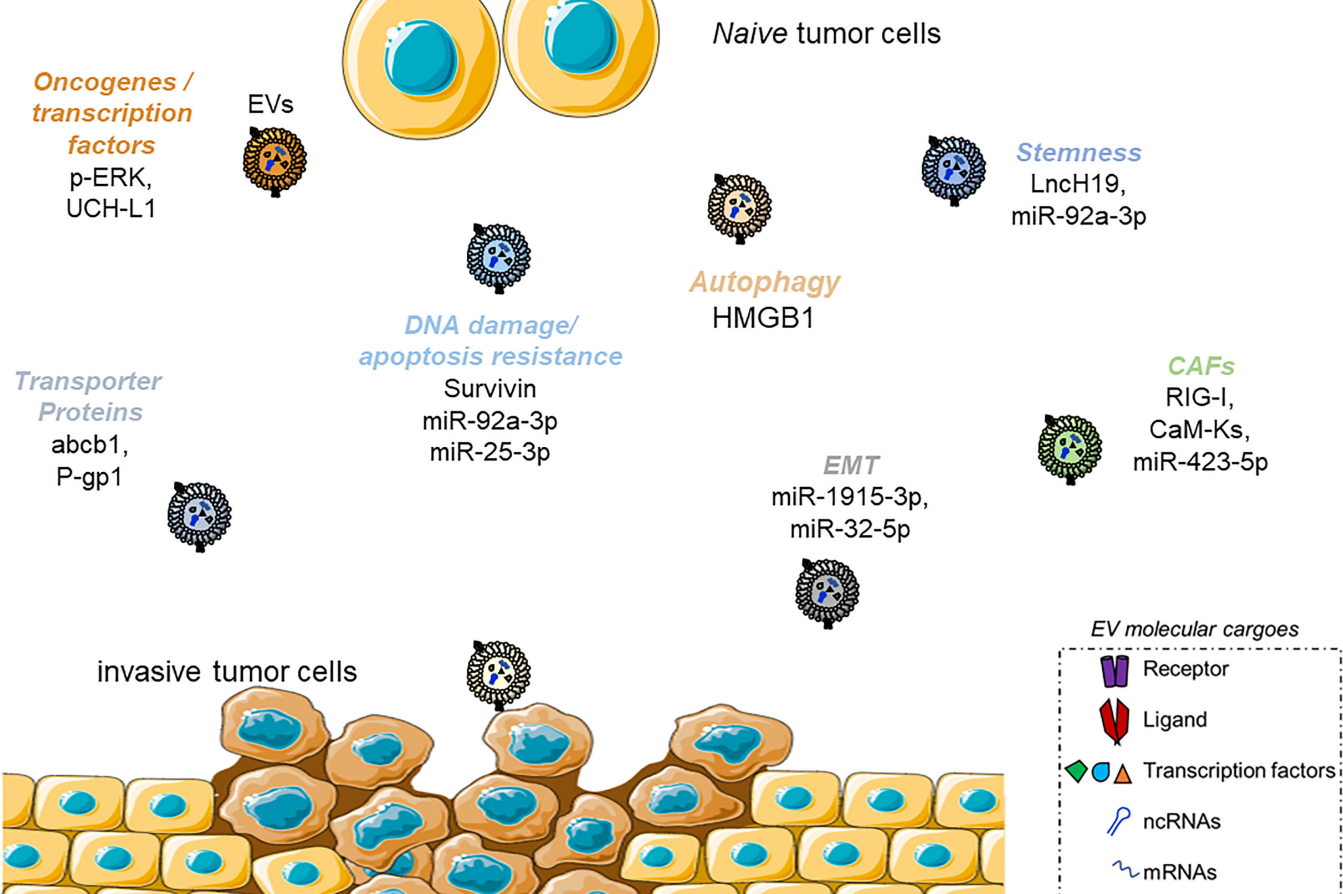
Scheme summarizing the mechanism of drug resistance activated by EVs. Examples of molecular players for each category, as described in **Figure 2**, are reported. Only molecules shuttled by EVs are reported.

In recent years, not only the tumor progression and growth but also the response to drugs and the outcome of antitumoral therapies have been associated with the specific effects of EVs, and precise pathways favoring tumor growth and facilitating metastasis have been described (132). For example, EVs are enriched in particular families of non-coding RNAs (microRNAs (miRNAs), long non-coding RNAs (lncRNAs), and circular RNAs (circRNAs)] involved in the epigenetic regulation of gene expression (133). *MALAT1*, a long-non-coding RNA associated with tumor metastasis and invasion in lung cancer and hepatocellular carcinoma (HCC), has also been found to be enriched in EVs from cervical carcinomas and breast cancer cells (134–136). LncRNA* TUC339* is responsible for regulating the proliferation and adhesion of HCC and is shuttled in EVs (137, 138). Through the shuttling of miRNAs, tumor cells can also acquire insensitiveness toward drugs. Yoshida *et al.* described in human biopsies of patients suffering from osteosarcoma (OS) an upregulation of miR-25-3p, negatively correlated with the clinical outcome (139). The same group demonstrated that miR-25-3p silences the Dickkopf WNT signaling pathway inhibitor 3 (DKK3) gene, thus supporting *in vitro* cancer growth and resistance to different chemotherapeutics [methotrexate, cisplatin, doxorubicin, and docetaxel (DOC)]. Similar effects have resembled after direct DKK3 silencing. Finally, miR-25-3p was found in cancer cell-derived EVs. In a similar study, Pan et al. confirmed the clinical relevance of EV-mediated drug resistance in OS patients (140). The authors revealed that circulating EVs from 43 OS patients presented the overexpression of the circular RNA circRNA103801 compared to healthy subjects. This EV cargo showed a prognostic value for patients, having an inverse correlation with the overall survival. The authors further investigated that the overexpression of circRNA103801 in human OS cell line MG63 conferred resistance to cisplatin and cells released EVs enriched in the same circRNA. The uptake of these EVs from *naïve* MG63 and U2OS cells increased the resistance to cisplatin, upregulating the expression of P-gp and multidrug resistance protein 1 (MRP1). Takahashi et al. showed that HCC protects from sorafenib-induced apoptosis and cytotoxicity through the release of EVs enriched in *Linc-*ROR, *via* a TGFβ-CD133 axis (33). Exosomal miR-222 is responsible for the resistance to tamoxifen in MCF7 cells, suppressing p27 and estrogen receptor (ER) alpha expression (141). In the next paragraph, we will deeply focus on some EV-based mechanisms specifically interfering with some of the most important chemotherapeutics used for fighting cancers (**Figure 4**).

### Platin (Platinum-Based Drug) Resistance by EV Molecular Cargoes

Platins are coordination complexes of platinum having a mainly alkylating activity on DNA, acting as intrastrand, interstrand, or DNA-protein crosslinks (142). An interesting study from Weinman *et al.* clarifies the correlation between EVs and drug resistance in a spontaneous canine model of OS (143). Based on the lag time between amputation and the start of adjuvant carboplatin treatment (good, disease-free interval >300 days; poor, disease-free interval <100 days), the animals have been divided into two cohorts and the protein profile of circulating EVs was run by mass spectrometry. The proteomic profile identified that tetranectin (TN) is decreased in the poor prognosis group and can be used as the most reliable biomarker. TN, a member of the C-type lectin family, shows a proteolytic activity. In the bone, TN has a crucial role in mineralization during osteogenesis and in extracellular matrix stiffness. Accordingly, the genetic loss of TN causes extracellular matrix softening and skeletal deformities (144). In a mouse model of CRC, LncH19-enriched EVs have been revealed to be promoters of oxaliplatin resistance. LncH19-EVs are released by CAFs and uptake by CRC cells SW480. Inside target cells, H19 activated the β-catenin pathway *via* acting as a competing endogenous RNA sponge for miR-141, an inhibitor of the cancer stemness. The overexpression of H19 was also confirmed in CRC patient samples at different tumor node metastasis stages (145). Lin and colleagues found that carnitine palmitoyltransferase 1A (CPT1A) was more highly expressed in colon cancer tissues than in noncancerous tissues and confirmed that CPT1A was increased by oxaliplatin stimulation in human colon cancer cell lines HCT116 and SW480. Silencing RNA could reverse the sensitivity of drug-resistant colon cancer cells to oxaliplatin (146). An elegant study showed in CRC cells the role of the antisense-RNA PGM5-AS1 in oxaliplatin resistance. Comparing tumor biopsies and perineoplastic tissues from patients, the authors found PGM5-AS1 as the second most downregulated ncRNA. In oxaliplatin-resistant SW480 cells, the downregulation of PGM5-AS1 is accompanied by the upregulation of the transcription repressor growth factor independent 1B (GFI1B). Further experiments demonstrated that GFI1B suppresses the expression of non-coding antisense RNA PGM5-AS1, which acts as a sponge for has-miR-423-5p to upregulate the expression of Nucleoside Diphosphate Kinase 1, NME1, and EVs can be involved in the intercellular exchange of the member of these pathways, contributing to resistance to oxaliplatin (147). Another miRNA involved in CRC was reported by Xiao *et al.* They found that the exosomal delivery of miR-1915-3p can improve the chemotherapeutic efficacy of oxaliplatin in CRC cells by suppressing the expression of 6-phosphofructo-2-kinase/fructose-2,6-biphosphatase 3 (PFKFB3) and ubiquitin carboxyl-terminal hydrolase 2 (USP2) and inducing the expression of E-cadherin (148). Another study showed the involvement of cirRNAs in the EV-based resistance to the FOLFOX regimen (oxaliplatin, 5-fluorouracil, folic acid) in CRC patients (149). A microarray profiling of exosomal circRNAs in FOLFOX-resistant HCT116 colon cancer cells identified 105 upregulated and 34 downregulated circRNAs compared to parental cells, with hsa_circ_0000338 being the most upregulated. Finally, the drug resistance can be transferred from resistant cells into sensitive cells *via* the uptake of exosomes. As reported in the previous paragraph, cancer cells can take advantage of CAF to acquire chemoresistance. Indeed, it has been shown that EVs from CAFs sustain chemo- and radio-resistance to the cisplatin of breast cancer MDA-MB-231 cells by activating retinoic acid-inducible gene I (RIG-I), the signal transducer and activator of transcription (STAT) 1, and NOTCH3 pathways (150). These studies are summarized in **Table 1**.

**Table 1:** Resistance to platins mediated by EVs.

Drug	Cancer Type	EV Cargo	Effects	Refs
Cisplatin	Osteosarcoma patients and *in vitro*	miR-25-3p	Inhibition of DKK3	(139)
Carboplatin	Osteosarcoma, spontaneous canine tumor	Tetranectin	Unreported	(143)
Oxaliplatin	CRC, *in vivo* and *in vitro*	LncH19-EVs	Sponge for miR-141 and β-catenin activation	(145)
Oxaliplatin	CRC *in vivo*	CPT1A	Unreported	(146)
Oxaliplatin	CRC *in vitro*	PGM5-AS1	Sponge for miR-423-5p and upregulation of NME1	(147)
Oxaliplatin	CRC *in vitro*	miR-1915-3p	Suppression of PFKFB3 and USP2	(148)
Oxaliplatin (FOLFOX)	CRC patients, *in vitro*	circ_0000338	Unreported	(149)
Cisplatin	Breast cancer, *in vitro*	EVs from CAF, unreported	Activation of RIG-I, STAT1, and NOTCH3	(150)

### Antimetabolite Resistance by EV Molecular Cargoes

5-fluorouracil (5-FU) is an antimetabolite working as a thymidylate synthase inhibitor, finally depleting the cells of the pyrimidine thymidylate, a nucleotide pivotal for DNA replication (151). Although 5-FU is the first-choice drug for cancer treatment, its efficiency is limited by the acquisition of an innate or acquired resistance. Zhao *et al.* showed that circ_0000338 is upregulated in 5-FU-resistant CRC both *in vitro* and *in vivo* as well as in CRC patients (152). The authors revealed that exosomes containing circ_0000338 are delivered from resistant to sensitive cells and confirmed that circulating EVs in CRC patients were enriched in circ_0000338. Further experiments revealed miR-217 and miR-485-3p as the target miRNAs of circ_0000338. Particularly, miR-217 induces tumor suppression by targeting downstream genes such as astrocyte elevated gene-1 (AEG-1), mitogen-activated protein kinase (MAPK), and zinc finger E-box binding homeobox 1 (ZEB1). On the other part, miR-485-3p counteracts CRC development, inhibiting the targeting protein for Xklp2 (TPX2). Runbi Ji *et al.* showed that EVs from human mesenchymal stem cells (MSCs) isolated from the umbilical cord promoted the 5-FU resistance of gastric cancer both *in vitro* and *in vivo* (153). In fact, the exposition to MSC-EVs induced in gastric cancer cells HGC-27, MGC-803, and SGC-7901 the activation of calcium/calmodulin-dependent protein kinases (CaM-Ks) and Raf/MEK/ERK kinase pathways, culminating in the upregulation of MDR, multidrug resistance-associated protein (MRP) and lung resistance-related protein (LRP), and finally, insensitiveness to apoptosis induced by 5-FU. In another study, miR-92a-3p expression in the circulating EVs of CRC patients has been demonstrated to be correlated with metastasis to the liver and chemoresistance to 5-FU (154). The main players in this context were CAFs, exhibiting the upregulation of miR-92a-3p, compared to normal fibroblasts, delivered in the surrounding by EVs. Once uptaken by cancer cells, miR-92a-3p-EVs induced the stemness, EMT, metastatization, and 5-FU resistance of cancer cells both *in vitro* and *in vivo*. Finally, F-Box and WD Repeat Domain Containing 7 (FBXW7) and Modulator of Apoptosis 1 (MOAP1) were identified as the main targets of miR-92a-3p. Consistently, CRC biopsies resulted to being enriched in miR-92a-3p and depleted in FBXW7 and MOAP1. Colon cancer cells exposed to 5-FU are also able to increase angiogenesis (155). Upon exposition to 5-FU, cancer cells HCT-15 released EVs enriched in growth/differentiation factor 15 (GDF15), which binds the TGF-βIII receptor. The activation of the receptor induces the suppression of Smad signaling and the upregulation of periostin in endothelial cells, culminating in the increase of angiogenesis. In HCC, Fu and colleagues found that HCC cells Bel7402 resistant to 5-FU produce EVs enriched in miR-32-5p shuttled to sensitive parental cells, in which it induces a decrease in PTEN and the activation of the PI3K/Akt, triggering EMT, angiogenesis, and finally, chemoresistance (156). Lastly, the relevance of miR-32-5p and PTEN in human HCC samples was investigated, confirming a negative correlation between them. This evidence is summarized in **Table 2**.

**Table 2:** Resistance to antimetabolites mediated by EVS.

Drug	Cancer Type	EV Cargo	Effects	Refs
5-FU	CRC patients and *in vitro*	circ_0000338	Repression of miR-217 and miR-485-3p upregulation of AEG-1, MAPK, ZEB1, and TPX2	(152)
5-FU	Gastric cancer patients and *in vitro*	EVs from MSCs	Upregulation of CaM-Ks, ERK, MDR, MRP, and LRP	(153)
5-FU	CRC patients *in vitro*	miR-92a-3p from CAFS	Downregulation of FBXW7 and MOAP1	(154)
5-FU	CRC *in vitro*	GDF15	Suppression of Smad and upregulation of periostin in target endothelial cells	(155)
5-FU	HCC patients and *in vitro*	miR-32-5p	Downregulation of PTEN and activation of EMT	(156)

5-FU, 5-fluorouracile.

### Doxorubicin Resistance by EV Molecular Cargoes

Doxorubicin (DXR), also known as Adriamycin (ADR), is an anthracycline antibiotic with antineoplastic activity, isolated from the bacterium *Streptomyces peucetius* var. *caesius*, acting as intercalating base pairs in the DNA helix (157). Additionally, DXR inhibits topoisomerase II. DXR also forms oxygen free radicals, resulting in cytotoxicity secondary to the lipid peroxidation of cell membrane lipids. Primary human OS cells MG63 treated with DXR increased their expression of P-gp1. Consistently, EVs from DXR-treated MG63 presented with higher levels of both the ABCB1 transcript and P-gp-encoded protein expression, which can be transferred to untreated MG63 cells, conferring their drug resistance (158).

Breast cancer cells MCF7 treated with ADR showed a high level of UCH-L1 and phospho-ERK, involved in the overexpression of ABCB1, compared to control cells. EVs from ADR-resistant MCF7 conferred to *naïve* MCF7 cells reduced sensitivity to ADR and increased p-ERK and P-gp1 levels (159). Interestingly, circulating EVs from breast cancer patients were positive for UCH-L1 and show an inverse correlation with response to treatment. MCF-7 exposed to ADR and DOC increased both cellular and exosomal miR-222, an inhibitor of phosphatase and tensin homolog (PTEN) gene, a tumor suppressor that negatively regulates the synthesis of phosphatidylinositol trisphosphate and the Akt signaling (141). Upon the uptake of these EVs, interstitial M2 macrophages underwent activation and polarization to support cancer cells. Accordingly, miR-222 has also been found in EVs from the plasma and tissue of chemoresistant patients (141). The overexpression of glutathione-S-transferase P1 (GSTP1, a phase II-metabolizing enzyme that detoxifies chemicals by conjugating with glutathione) is a described tool for cancer cells for counteracting chemotherapeutics like ADR. Yang *et al.* found that this enzyme is present in EVs from ADR-resistant MCF7 and, accordingly, in the sera of chemoresistant patients (160). A study conducted on HCC cells reported the role of long non-coding RNA linc-VLDLR in resistance toward DXR. Linc-VLDLR promoted the expression of the PCNA and ABCG2 genes, and the EV-mediated transfer of linc-VLDLR can result in the chemoresistance of HCC (161). **Table 3** summarizes these studies.

**Table 3:** Resistance to doxorubicin (DXR) mediated by EVS.

Drug	Cancer Type	EV Cargo	Effects	Refs
DXR	Osteosarcoma, *in vitro*	P-gp1	Transfer of mRNA and protein of MDR in sensitive cells	(158)
DXR	Breast cancer, patients and *in vitro*	UCH-L1 and p-ERK	Increase of ABCB1	(159)
DXR	Breast cancer, patients and *in vitro*	miR-222	Suppression of PTEN in M2 macrophage	(141)
DXR	Breast cancer patients and *in vitro*	GSTP1	Enhancement of detoxification pathway	(160)
DXR	HCC *in vitro*	Linc-VLDLR	Upregulation of PCNA and ABCG2	(161)

### Taxane Resistance by EV Molecular Cargoes

Taxanes are diterpenes firstly isolated from the plants of *Taxus* spp. Taxanes work as microtubule-stabilizing drugs, inhibiting the depolymerization of microtubules during cell division (162). PTX, DOC, and cabazitaxel are widely used for the treatment of many different cancers. Exposure to the DOC of MCF7 cells induced the expression of P-gp1. Moreover, EVs from DOC-treated MCF-7 expressed higher levels of P-gp compared to EVs from *naïve* MCF-7, and the incubation with DOC-MCF7 EVs reduced the cell apoptosis of *naïve* MCF-7 (163). In another study, breast cancer cells MDA-MB-231 exposed to PTX specifically released EVs enriched in survivin, an inhibitor of apoptosis (164). The survivin-enriched EVs exerted protective effects on drug-sensitive fibroblasts and SKBR3 cells when exposed to PTX. Shan *et al.* described the role of CAF-EVs in the taxane resistance of prostate cancer cells. CAFs released EVs enriched in miR-423-5p, which is internalized inside prostate cancer cells LNCAP, 22RV-1, and C4 suppressing GREM2 [encoding for the gremlin2 protein inhibitor of bone morphogenetic protein (BMP) family members] and increasing TGF-β, overall leading to a reduced sensitivity to taxanes (165). These results are reported in **Table 4**.

**Table 4:** Resistance to taxanes mediated by EVS.

Drug	Cancer Type	EV Cargo	Effects	Refs
Docetaxel	Breast cancer, *in vitro*	P-gp	Expression of functional P-gp	(163)
Paclitaxel	Breast cancer, *in vitro*	Survivin	Inhibition of apoptosis	(164)
Taxanes	Prostate cancer, *in vitro*	miR-423-5p from CAF-EVs	Inhibition of GREM2 and increase of TGF-β in cancer cells	(165)

### Biological Drugs by EV Molecular Cargoes

Monoclonal antibodies are the newest frontier of anticancer drugs. A deeper knowledge of cancer biology and molecular profile allows to precisely target a specific member of pivotal pathways for cancer growth. Unfortunately, tumors can find a strategy to also counteract these agents. Cetuximab is a humanized mouse monoclonal antibody against the EGFR. Zhang *et al.* showed that EVs derived from cetuximab-resistant RKO colon cancer cells induced cetuximab resistance in cetuximab-sensitive Caco-2 cells. RKO cells and RKO-EVs resulted in depleted PTEN and enriched phospho-Akt, and the EV effects were abrogated by the Akt inhibitor LY294002 (166). In another study, circulating EVs from patients suffering from CRC have been exploited as a predictive biomarker for the response to cetuximab (42). In particular, circulating EVs from metastatic and chemoresistant subjects resulted in enriched lncRNA urothelial carcinoma-associated 1 (UCA1). *In vitro* experiments revealed that exosomes from cetuximab-resistant Caco-2 cells can transmit drug UCA1 and resistance to sensitive parental cells. Epidermal growth factor receptor 2 (HER2)-positive tumors can be targetable with the monoclonal antibody trastuzumab. Disappointingly, tumor cells can neutralize trastuzumab by EV release, *via* a decoy-like system. HER2-positive breast cancer cells BT474 and SKBR3 release HER2-positive EVs able to bind trastuzumab, while EVs from triple-negative cells MDA-MD-231 do not. On this basis, SKBR3 cells treated with autologous EVs were less sensitive to the effect of trastuzumab since EVs sequester trastuzumab, reducing the efficacy of the chemotherapy against the primary tumor (167). Finally, circulating EVs from HER2-positive breast cancer patients at an early stage showed lower binding to trastuzumab compared to EVs from patients with advanced disease. Rituximab is a monoclonal antibody against CD20, a standard in the management of malignant B-cell lymphoma (168). Aung *et al.* showed that leukemic cells released CD20-enriched EVs intercepting rituximab, thus protecting cancer cells from the complement-dependent cytolysis induced by rituximab (169). Lubin and colleagues reported that neuroblastoma cells released programmed death-ligand 1 (PD-L1)-EVs that bind to PD-1 on the surfaces of cytotoxic T cells, preventing the targeting of tumor cells and finally allowing immune evasion (170). Other authors described that the response to pembrolizumab (an anti-PD-1 antibody) in patients suffering from melanoma can be reduced by EVs (171). After treatment with pembrolizumab, melanoma cells released EVs enriched in PD-L1, which suppresses the proliferation of cytotoxic T cells and facilitates the immune evasion of tumor cells, counteracting the efficacy of pembrolizumab. **Table 5** summarizes these studies.

**Table 5:** Resistance to biological drugs mediated by EVS.

Drug	Cancer Type	EV Cargo	Effects	Refs
Cetuximab	CRC, *in vitro*	p-Akt	Depletion of PTEN	(166)
Cetuximab	CRC patients and *in vitro*	UCA1	Unreported	(42)
Trastuzumab	Breast cancer patients and *in vitro*	HER2	Neutralizing trastuzumab	(167)
Rituximab	B-cell lymphoma, *in vitro* and patients	CD20	Neutralizing rituximab	(168, 169)
Pembrolizumab	Melanoma patients and *in vitro*	PD-L1	Neutralizing pembrolizumab	(171)

## Discussion

EVs are nowadays reported to be responsible for sustaining many aspects of tumor biology. Cancer recurrence and metastatization are the main clinical challenges to offer to patients a perspective of a free-disease lifespan or at least a lifetime with a steadied cancer. Resistance to therapies is one of the causative agents of those challenges. Many mechanisms are described to drive chemoresistance, and, of note, they frequently overlap, making it virtually impossible to counteract once activated. EVs are recently indicated as a further mechanism supporting chemoresistance. By the means of EVs, insensitive cancer cells can educate sensitive cognate cells, shuttling directly a functional molecular apparatus. On this basis, EVs gain clinical interest as a manageable biomarker of cancer aggressiveness and predisposition to chemoresistance, becoming a promising liquid biopsy.

Moreover, EVs also offer a new strategy to fight cancer *via* the inhibition of the release or the interaction of EVs with target cells. Unfortunately, some issues dampen the use of EVs in clinical and therapeutic management. In fact, while in basic and preclinical studies, the key involvement of EVs is incontrovertible, these results are dampened in patients and not completely reproducible, mostly comparing *in vitro* studies with human trials. A reason for that can be the use of different procedures adopted to isolate EVs since a universal consensus is still lacking on this aspect. It is nowadays reported that the specific isolating procedures can enrich protein contamination (i.e., lipoproteins or protein aggregates), and certain EV subpopulations, in turn, selecting a particular molecular cargo not strictly linked to the real biological condition, making it hard to extrapolate a comprehensive and objective interpretation.

Nevertheless, many authors are exploiting the use of natural or modified EVs as a drug delivery system. A successful and effective encapsulation of chemotherapeutics has been reported for DXR, cisplatin, and methotrexate in EVs from lung (human A549 cells), hepatocarcinoma (murine H22 cells), and breast (human MCF-7 cells) cancer cells. The efficacy has been demonstrated *in vitro* in animal models and in a clinical trial (patients suffering from stage IV lung carcinoma) (172). Paclitaxel was also loaded in EVs from human prostate cancer cells (LNCaP and PC-3 cells) (173). Murine macrophage RAW 264.7 cells were tested as a source of EVs for loading DXR targeting lung and colon (both *in vitro* and in animal models) cancers (174, 175). Similarly, EVs from RAW 264.7 packaged with DXR were effective in H22 tumor-bearing mice (144). Primary murine osteoblast-EVs have been loaded with dasatinib and successfully mitigated exacerbated osteolysis *in vivo* (18).

This is a very stimulating and active field since EVs offer many advantages in drug administration compared to the classical or liposomal formulation. The naturally occurring EV composition can confer very selective tropism to a specific tissue or cell, as well as present a higher biologic activity due to the ability to convey complex molecular machinery to the targets sustaining the required therapeutic effect. Moreover, natural EVs can be engineered for acquiring further or better properties (175).

As discussed above, several issues still curb the possibility to produce EVs for therapeutic use, mainly because the option to produce EVs under Good Manufacturing Process conditions is still lacking, although many efforts are leading in this direction. In this sense, all the key unit operation and process steps are under consideration for standardization and assessment for EV safety and de-risking, considering and not limited to the following: choice and characterization of the cell source, isolation methods, drug-loading methods (loading efficacy/cost ratio for large-scale production), the eradication of potential contaminants and impurities, best formulation, and the shelf life of final EV products. The recent case of a public safety notification on exosome products from the Food and Drug Administration for a group of patients in Nebraska, who have experienced adverse effects from the administration of improper EVs, is an exemplificative of the urgency to have regulatory monitoring about the use of EVs for human health (https://www.fda.gov/vaccines-blood-biologics/safety-availability-biologics/public-safety-notification-exosome-products). The increasing availability of new analytical techniques is predictable to provide new insights into the distinctiveness of EVs and may unlock the full potential of EVs for clinical management.

## Author Contributions

SP: Conceptualization, Literature Review, Visualization, Writing—Review and Editing. AV: Coordination, Writing—Original Draft. RS: Supervision, Writing—Review and Editing. AC: Conceptualization, Literature Searching and Critical Review, Writing—Original Draft. All authors have read and approved the final manuscript.

## Conflict of Interest

The authors declare that the research was conducted in the absence of any commercial or financial relationships that could be construed as a potential conflict of interest.

## Publisher’s Note

All claims expressed in this article are solely those of the authors and do not necessarily represent those of their affiliated organizations, or those of the publisher, the editors and the reviewers. Any product that may be evaluated in this article, or claim that may be made by its manufacturer, is not guaranteed or endorsed by the publisher.

## References

[1] Van Niel G D’Angelo G Raposo G. Shedding Light on the Cell Biology of Extracellular Vesicles. Nat Rev Mol Cell Biol (2018) 19:213–28. doi: 10.1038/nrm.2017.125 29339798

[2] Couch Y Buzàs EI Di Vizio D Gho YS Harrison P Hill AF. A Brief History of Nearly EV-Erything - The Rise and Rise of Extracellular Vesicles. J Extracell Vesicles (2021) 10:e12144–56. doi: 10.1002/jev2.12144 PMC8681215 34919343

[3] O’Brien K Breyne K Ughetto S Laurent LC Breakefield XO. RNA Delivery by Extracellular Vesicles in Mammalian Cells and its Applications. Nat Rev Mol Cell Biol (2020) 21:585–606. doi: 10.1038/s41580-020-0251-y 32457507 PMC7249041

[4] Loftus A Cappariello A George C Ucci A Shefferd K Green A. Extracellular Vesicles From Osteotropic Breast Cancer Cells Affect Bone Resident Cells. J Bone Miner Res (2020) 35:396–412. doi: 10.1002/jbmr.3891 31610048

[5] Colombo M Raposo G Théry C. Biogenesis, Secretion, and Intercellular Interactions of Exosomes and Other Extracellular Vesicles. Annu Rev Cell Dev Biol (2014) 30:255–89. doi: 10.1146/annurev-cellbio-101512-122326 25288114

[6] Théry C Witwer KW Aikawa E Alcaraz MJ Anderson JD Andriantsitohaina R. Minimal Information for Studies of Extracellular Vesicles 2018 (MISEV2018): A Position Statement of the International Society for Extracellular Vesicles and Update of the MISEV2014 Guidelines. J Extracell Vesicles (2018) 7:1535750–97. doi: 10.1080/20013078.2018.1535750 PMC6322352 30637094

[7] Katzmann DJ Babst M Emr SD. Ubiquitin-Dependent Sorting Into the Multivesicular Body Pathway Requires the Function of a Conserved Endosomal Protein Sorting Complex, ESCRT-I. Cell (2001) 106:145–55. doi: 10.1016/S0092-8674(01)00434-2 11511343

[8] Colombo M Moita C van Niel G Kowal J Vigneron J Benaroch P. Analysis of ESCRT Functions in Exosome Biogenesis, Composition and Secretion Highlights the Heterogeneity of Extracellular Vesicles. J Cell Sci (2013) 126:5553–65. doi: 10.1242/jcs.128868 24105262

[9] Trajkovic K Hsu C Chiantia S Rajendran L Wenzel D Wieland F. Ceramide Triggers Budding of Exosome Vesicles Into Multivesicular Endosomes. Science (2008) 319:1244–7. doi: 10.1126/science.1153124 18309083

[10] Muralidharan-Chari V Clancy J Plou C Romao M Chavrier P Raposo G. ARF6-Regulated Shedding of Tumor Cell-Derived Plasma Membrane Microvesicles. Curr Biol (2009) 19:1875–85. doi: 10.1016/j.cub.2009.09.059 PMC3150487 19896381

[11] Atkin-Smith GK Tixeira R Paone S Mathivanan S Collins C Liem M. A Novel Mechanism of Generating Extracellular Vesicles During Apoptosis *via* a Beads-on-a-String Membrane Structure. Nat Commun (2015) 6:7439–49. doi: 10.1038/ncomms8439 PMC4490561 26074490

[12] Parolini I Federici C Raggi C Lugini L Palleschi S De Milito A. Microenvironmental pH is a Key Factor for Exosome Traffic in Tumor Cells. J Biol Chem (2009) 284:34211–22. doi: 10.1074/jbc.M109.041152 PMC2797191 19801663

[13] Nanbo A Kawanishi E Yoshida R Yoshiyama H. Exosomes Derived From Epstein-Barr Virus-Infected Cells Are Internalized *via* Caveola-Dependent Endocytosis and Promote Phenotypic Modulation in Target Cells. J Virol (2013) 87:10334–47. doi: 10.1128/JVI.01310-13 PMC3753980 23864627

[14] Fitzner D Schnaars M van Rossum D Krishnamoorthy G Dibaj P Bakhti M. Selective Transfer of Exosomes From Oligodendrocytes to Microglia by Macropinocytosis. J Cell Sci (2011) 124:447–58. doi: 10.1242/jcs.074088 21242314

[15] Feng D Zhao W-L Ye Y-Y Bai X-C Liu R-Q Chang L-F. Cellular Internalization of Exosomes Occurs Through Phagocytosis. Traffic (2010) 11:675–87. doi: 10.1111/j.1600-0854.2010.01041.x 20136776

[16] Kholia S Ranghino A Garnieri P Lopatina T Deregibus MC Rispoli P. Extracellular Vesicles as New Players in Angiogenesis. Vascul Pharmacol (2016) 86:64–70. doi: 10.1016/j.vph.2016.03.005 27013016

[17] Conde-Vancells J Rodriguez-Suarez E Embade N Gil D Matthiesen R Valle M. Characterization and Comprehensive Proteome Profiling of Exosomes Secreted by Hepatocytes. J Proteome Res (2008) 7:5157–66. doi: 10.1021/pr8004887 PMC2696236 19367702

[18] Cappariello A Loftus A Muraca M Maurizi A Rucci N Teti A. Osteoblast-Derived Extracellular Vesicles Are Biological Tools for the Delivery of Active Molecules to Bone. J Bone Miner Res (2018) 33:517–33. doi: 10.1002/jbmr.3332 29091316

[19] McKay TB Hutcheon AEK Zieske JD Ciolino JB. Extracellular Vesicles Secreted by Corneal Epithelial Cells Promote Myofibroblast Differentiation. Cells (2020) 9:1080–94. doi: 10.3390/cells9051080 PMC7290736 32357574

[20] Aliotta JM Pereira M Sears EH Dooner MS Wen S Goldberg LR. Lung-Derived Exosome Uptake Into and Epigenetic Modulation of Marrow Progenitor/Stem and Differentiated Cells. J Extracell Vesicles (2015) 4:26166–79. doi: 10.3402/jev.v4.26166 PMC4575417 26385657

[21] Yang J Zheng XG Wu YL Wang AP Wang CH Chen WX. Intestinal Epithelial Cell-Derived Exosomes Package microRNA-23a-3p Alleviate Gut Damage After Ischemia/Reperfusion *via* Targeting MAP4K4. BioMed Pharmacother (2022) 149:112810–26. doi: 10.1016/j.biopha.2022.112810 35303564

[22] Alvarez C-S Badia J Bosch M Giménez R Baldomà L. Outer Membrane Vesicles and Soluble Factors Released by Probiotic Escherichia Coli Nissle 1917 and Commensal ECOR63 Enhance Barrier Function by Regulating Expression of Tight Junction Proteins in Intestinal Epithelial Cells. Front Microbiol (2016) 7:1981. doi: 10.3389/fmicb.2016.01981 28018313 PMC5156689

[23] Pieters BCHH Cappariello A van den Bosch MHJJ van Lent PLEMEM Teti A van de Loo FAJJ. Macrophage-Derived Extracellular Vesicles as Carriers of Alarmins and Their Potential Involvement in Bone Homeostasis. Front Immunol (2019) 10:1901. doi: 10.3389/fimmu.2019.01901 31440259 PMC6694442

[24] Sahu A Clemens ZJ Shinde SN Sivakumar S Pius A Bhatia A. Regulation of Aged Skeletal Muscle Regeneration by Circulating Extracellular Vesicles. Nat Aging (2021) 1:1148–61. doi: 10.1038/s43587-021-00143-2 PMC9165723 35665306

[25] Zhang ZG Chopp M. Exosomes in Stroke Pathogenesis and Therapy. J Clin Invest (2016) 126:1190–7. doi: 10.1172/JCI81133 PMC4811130 27035810

[26] Sferra R Pompili S Cappariello A Gaudio E Latella G Vetuschi A. Prolonged Chronic Consumption of a High Fat With Sucrose Diet Alters the Morphology of the Small Intestine. Int J Mol Sci (2021) 22:7280–94. doi: 10.3390/ijms22147280 PMC8303301 34298894

[27] Kumar A Sundaram K Mu J Dryden GW Sriwastva MK Lei C. High-Fat Diet-Induced Upregulation of Exosomal Phosphatidylcholine Contributes to Insulin Resistance. Nat Commun (2021) 12:213–27. doi: 10.1038/s41467-020-20500-w PMC7801461 33431899

[28] Wang W Li M Chen Z Xu L Chang M Wang K. Biogenesis and Function of Extracellular Vesicles in Pathophysiological Processes of Skeletal Muscle Atrophy. Biochem Pharmacol (2022) 198:114954–69. doi: 10.1016/j.bcp.2022.114954 35167807

[29] Gao XJ Li T Wei B Yan ZX Hu N Huang YJ. Bacterial Outer Membrane Vesicles From Dextran Sulfate Sodium–Induced Colitis Differentially Regulate Intestinal UDP-Glucuronosyltransferase 1A1 Partially Through Toll-Like Receptor 4/Mitogen-Activated Protein Kinase/Phosphatidylinositol 3-Kinase Pathway. Drug Metab Dispos (2018) 46:292–302. doi: 10.1124/DMD.117.079046 29311138

[30] Kang C Ban M Choi E-J Moon H-G Jeon J-S Kim D-K. Extracellular Vesicles Derived From Gut Microbiota, Especially Akkermansia Muciniphila, Protect the Progression of Dextran Sulfate Sodium-Induced Colitis. PLoS One (2013) 8:e76520–36. doi: 10.1371/journal.pone.0076520 PMC3811976 24204633

[31] Vetuschi A Battista N Pompili S Cappariello A Prete R Taticchi A. The Antiinflammatory and Antifibrotic Effect of Olive Phenols and Lactiplantibacillus Plantarum IMC513 in Dextran Sodium Sulfate–Induced Chronic Colitis. Nutrition (2022) 94:111511–28. doi: 10.1016/j.nut.2021.111511 34813981

[32] Wei ZX Xie GJ Mao X Zou XP Liao YJ Liu QS. Exosomes From Patients With Major Depression Cause Depressive-Like Behaviors in Mice With Involvement of miR-139-5p-Regulated Neurogenesis. Neuropsychopharmacol 2020 456 (2020) 45:1050–8. doi: 10.1038/s41386-020-0622-2 PMC7162931 31986519

[33] Takahashi K Yan IK Kogure T Haga H Patel T. Extracellular Vesicle-Mediated Transfer of Long non-Coding RNA ROR Modulates Chemosensitivity in Human Hepatocellular Cancer. FEBS Open Bio (2014) 4:458–67. doi: 10.1016/J.FOB.2014.04.007 PMC4050189 24918061

[34] Peinado H Alečković M Lavotshkin S Medicine, Matei I Costa-Silva B Moreno-Bueno G. Melanoma Exosomes Educate Bone Marrow Progenitor Cells Toward a Pro-Metastatic Phenotype Through MET. Nat Med (2012) 18:883–91. doi: 10.1038/nm.2753 PMC3645291 22635005

[35] Bigagli E Luceri C Guasti D Cinci L. Exosomes Secreted From Human Colon Cancer Cells Influence the Adhesion of Neighboring Metastatic Cells: Role of microRNA-210. Cancer Biol Ther (2016) 17:1062–9. doi: 10.1080/15384047.2016.1219815 PMC5079399 27611932

[36] Qiu J-J Lin X-J Tang X-Y Zheng T-T Lin Y-Y Hua K-Q. Exosomal Metastasis−Associated Lung Adenocarcinoma Transcript 1 Promotes Angiogenesis and Predicts Poor Prognosis in Epithelial Ovarian Cancer. Int J Biol Sci (2018) 14:1960–73. doi: 10.7150/ijbs.28048 PMC6299373 30585260

[37] Ucci A Cappariello A Ponzetti M Tennant F Loftus AEP Shefferd K. Anti-Osteoblastogenic, Pro-Inflammatory and Pro-Angiogenic Effect of Extracellular Vesicles Isolated From the Human Osteosarcoma Cell Line MNNG/HOS. Bone (2021) 153:116130–47. doi: 10.1016/j.bone.2021.116130 34329816

[38] Cappariello A Rucci N. Extracellular Vesicles in Bone Tumors: How to Seed in the Surroundings Molecular Information for Malignant Transformation and Progression. Front Oncol (2021) 11:722922. doi: 10.3389/fonc.2021.722922 34616676 PMC8488258

[39] Hanahan D Weinberg RA. Hallmarks of Cancer: The Next Generation. Cell (2011) 144:646–74. doi: 10.1016/j.cell.2011.02.013 21376230

[40] Qu J-L Qu X-J Zhao M-F Teng Y-E Zhang Y Hou K-Z. Gastric Cancer Exosomes Promote Tumour Cell Proliferation Through PI3K/Akt and MAPK/ERK Activation. Dig Liver Dis (2009) 41:875–80. doi: 10.1016/j.dld.2009.04.006 19473897

[41] Sun L-H Tian D Yang Z-C Li J-L. Exosomal miR-21 Promotes Proliferation, Invasion and Therapy Resistance of Colon Adenocarcinoma Cells Through its Target PDCD4. Sci Rep (2020) 10:8271–84. doi: 10.1038/s41598-020-65207-6 PMC7237414 32427870

[42] Yang YN Zhang R Du JW Yuan HH Li YJ Wei XL. Predictive Role of UCA1-Containing Exosomes in Cetuximab-Resistant Colorectal Cancer 11 Medical and Health Sciences 1112 Oncology and Carcinogenesis. Cancer Cell Int (2018) 18:1–11. doi: 10.1186/S12935-018-0660-6/FIGURES/5 30377411 PMC6196422

[43] Wang J Hendrix A Hernot S Lemaire M De Bruyne E Van Valckenborgh E. Bone Marrow Stromal Cell–Derived Exosomes as Communicators in Drug Resistance in Multiple Myeloma Cells. Blood (2014) 124:555–66. doi: 10.1182/blood-2014-03-562439 24928860

[44] Zheng H-C. The Molecular Mechanisms of Chemoresistance in Cancers. Oncotarget (2017) 8:59950–64. doi: 10.18632/oncotarget.19048 PMC5601792 28938696

[45] Khan S Jutzy JMS Valenzuela MMA Turay D Aspe JR Ashok A. Plasma-Derived Exosomal Survivin, a Plausible Biomarker for Early Detection of Prostate Cancer. PLoS One (2012) 7:e46737–49. doi: 10.1371/journal.pone.0046737 PMC3473028 23091600

[46] Koga K Matsumoto K Akiyoshi T Kubo M Yamanaka N Tasaki A. Purification, Characterization and Biological Significance of Tumor-Derived Exosomes. Anticancer Res (2005) 25:3703–7. 16302729

[47] Yang L Wu X-H Wang D Luo C-L Chen L-X. Bladder Cancer Cell-Derived Exosomes Inhibit Tumor Cell Apoptosis and Induce Cell Proliferation *In Vitro* . Mol Med Rep (2013) 8:1272–8. doi: 10.3892/mmr.2013.1634 23969721

[48] Qu J-L Qu X-J Qu J-L Qu X-J Zhao M-F Teng Y-E. The Role of Cbl Family of Ubiquitin Ligases in Gastric Cancer Exosome-Induced Apoptosis of Jurkat T Cells. Acta Oncol (Madr) (2009) 48:1173–80. doi: 10.3109/02841860903032817 19863226

[49] Gutkin A Uziel O Beery E Nordenberg J Pinchasi M Goldvaser H. Tumor Cells Derived Exosomes Contain hTERT mRNA and Transform Nonmalignant Fibroblasts Into Telomerase Positive Cells. Oncotarget (2016) 7:59173–88. doi: 10.18632/oncotarget.10384 PMC5312303 27385095

[50] Ma S McGuire MH Mangala LS Lee S Stur E Hu W. Gain-Of-Function P53 Protein Transferred *via* Small Extracellular Vesicles Promotes Conversion of Fibroblasts to a Cancer-Associated Phenotype. Cell Rep (2021) 34:108726–47. doi: 10.1016/j.celrep.2021.108726 PMC7957825 33567287

[51] Kalra H Gangoda L Fonseka P Chitti SV Liem M Keerthikumar S. Extracellular Vesicles Containing Oncogenic Mutant β-Catenin Activate Wnt Signalling Pathway in the Recipient Cells. J Extracell Vesicles (2019) 8:347–60. doi: 10.1080/20013078.2019.1690217/SUPPL_FILE/ZJEV_A_1690217_SM8326.ZIP PMC6883417 31819794

[52] Huang M Lei Y Zhong Y Chung C Wang M Hu M. New Insights Into the Regulatory Roles of Extracellular Vesicles in Tumor Angiogenesis and Their Clinical Implications. Front Cell Dev Biol (2021) 9:791882/BIBTEX. doi: 10.3389/FCELL.2021.791882/BIBTEX 34966744 PMC8710745

[53] Adem B Vieira PF Melo SA. Decoding the Biology of Exosomes in Metastasis. Trends Cancer (2020) 6:20–30. doi: 10.1016/j.trecan.2019.11.007 31952777

[54] Gottesman MM Lavi O Hall MD Gillet J-P. Toward a Better Understanding of the Complexity of Cancer Drug Resistance. Annu Rev Pharmacol Toxicol (2016) 56:85–102. doi: 10.1146/annurev-pharmtox-010715-103111 26514196

[55] Ouellette MM Zhou S Yan Y. Cell Signaling Pathways That Promote Radioresistance of Cancer Cells. Diagnostics (Basel Switzerland) (2022) 12:656–70. doi: 10.3390/DIAGNOSTICS12030656 PMC8947583 35328212

[56] Sferra R Pompili S Festuccia C Marampon F Gravina GL Ventura L. The Possible Prognostic Role of Histone Deacetylase and Transforming Growth Factor β/Smad Signaling in High Grade Gliomas Treated by Radio-Chemotherapy: A Preliminary Immunohistochemical Study. Eur J Histochem (2017) 61:96–105. doi: 10.4081/ejh.2017.2732 PMC5439439 28735518

[57] Noonan KE Beck C Holzmayer TA Chin JE Wunder JS Andrulis IL. Quantitative Analysis of MDR1 (Multidrug Resistance) Gene Expression in Human Tumors by Polymerase Chain Reaction. Proc Natl Acad Sci (1990) 87:7160–4. doi: 10.1073/pnas.87.18.7160 PMC54703 1976252

[58] Grant CE Gao M DeGorter MK Cole SPC Deeley RG. Structural Determinants of Substrate Specificity Differences Between Human Multidrug Resistance Protein (MRP) 1 (ABCC1) and MRP3 (Abcc3). Drug Metab Dispos (2008) 36:2571–81. doi: 10.1124/dmd.108.022491 18775981

[59] Deeley RG Cole SPC. Substrate Recognition and Transport by Multidrug Resistance Protein 1 (ABCC1). FEBS Lett (2006) 580:1103–11. doi: 10.1016/J.FEBSLET.2005.12.036 16387301

[60] Austin Doyle L Yang W Abruzzo LV Krogmann T Gao Y Rishi AK. A Multidrug Resistance Transporter From Human MCF-7 Breast Cancer Cells. Proc Natl Acad Sci U S A (1998) 95:15665–70. doi: 10.1073/PNAS.95.26.15665 PMC28101 9861027

[61] Stein U Lage H Jordan A Walther W Bates SE Litman T. Impact of BCRP/MXR, MRP1 and MDR1/P-Glycoprotein on Thermoresistant Variants of Atypical and Classical Multidrug Resistant Cancer Cells. Int J Cancer (2002) 97:751–60. doi: 10.1002/ijc.10131 11857350

[62] Chang F-W Fan H-C Liu J-M Fan T-P Jing J Yang C-L. Estrogen Enhances the Expression of the Multidrug Transporter Gene ABCG2—Increasing Drug Resistance of Breast Cancer Cells Through Estrogen Receptors. Int J Mol Sci (2017) 18:163–77. doi: 10.3390/ijms18010163 PMC5297796 28098816

[63] Scheffer GL Wijngaard PLJ Flens MJ Izquierdo MA Slovak ML Pinedo HM. The Drug Resistance-Related Protein LRP is the Human Major Vault Protein. Nat Med (1995) 1:578–82. doi: 10.1038/nm0695-578 7585126

[64] Slovak ML Ho JP Cole SPC Deeley RG Greenberger L De Vries EGE. The LRP Gene Encoding a Major Vault Protein Associated With Drug Resistance Maps Proximal to MRP on Chromosome 16: Evidence That Chromosome Breakage Plays. AACR (1995) 55:4214–9. 7671223

[65] Xiao YS Zeng D Liang YK Wu Y Li MF Qi YZ. Major Vault Protein is a Direct Target of Notch1 Signaling and Contributes to Chemoresistance in Triple-Negative Breast Cancer Cells. Cancer Lett (2019) 440–441:156–67. doi: 10.1016/J.CANLET.2018.09.031 30336197

[66] Cordani M Oppici E Dando I Butturini E Dalla Pozza E Nadal-Serrano M. Mutant P53 Proteins Counteract Autophagic Mechanism Sensitizing Cancer Cells to mTOR Inhibition. Mol Oncol (2016) 10:1008–29. doi: 10.1016/j.molonc.2016.04.001 PMC5423176 27118659

[67] Gan Y Shi C Inge L Hibner M Balducci J Huang Y. Differential Roles of ERK and Akt Pathways in Regulation of EGFR-Mediated Signaling and Motility in Prostate Cancer Cells. Oncogene (2010) 29:4947–58. doi: 10.1038/onc.2010.240 20562913

[68] Zhao K Wang Q Wang Y Huang K Yang C Li Y. EGFR/c-Myc Axis Regulates Tgfβ/Hippo/Notch Pathway *via* Epigenetic Silencing miR-524 in Gliomas. Cancer Lett (2017) 406:12–21. doi: 10.1016/j.canlet.2017.07.022 28778566

[69] He C Li L Guan X Xiong L Miao X. Mutant P53 Gain of Function and Chemoresistance: The Role of Mutant P53 in Response to Clinical Chemotherapy. Chemotherapy (2017) 62:43–53. doi: 10.1159/000446361 27322648

[70] Enoch T Norbury C. Cellular Responses to DNA Damage: Cell-Cycle Checkpoints, Apoptosis and the Roles of P53 and ATM. Trends Biochem Sci (1995) 20:426–30. doi: 10.1016/S0968-0004(00)89093-3 8533157

[71] Mirzayans R Andrais B Scott A Murray D. New Insights Into P53 Signaling and Cancer Cell Response to DNA Damage: Implications for Cancer Therapy. J BioMed Biotechnol (2012) 2012:170325. doi: 10.1155/2012/170325 22911014 PMC3403320

[72] Roemer K. Mutant P53: Gain-Of-Function Oncoproteins and Wild-Type P53 Inactivators. Biol Chem (1999) 380:879–87. doi: 10.1515/BC.1999.108 10494837

[73] Willis A Jung EJ Wakefield T Chen X. Mutant P53 Exerts a Dominant Negative Effect by Preventing Wild-Type P53 From Binding to the Promoter of its Target Genes. Oncogene (2004) 23:2330–8. doi: 10.1038/sj.onc.1207396 14743206

[74] Monti P Campomenosi P Ciribilli Y Iannone R Inga A Abbondandolo A. Tumour P53 Mutations Exhibit Promoter Selective Dominance Over Wild Type P53. Oncogene (2002) 21:1641–8. doi: 10.1038/sj.onc.1205250 11896595

[75] Zinatizadeh MR Schock B Chalbatani GM Zarandi PK Jalali SA Miri SR. The Nuclear Factor Kappa B (NF-Kb) Signaling in Cancer Development and Immune Diseases. Genes Dis (2021) 8:287–97. doi: 10.1016/J.GENDIS.2020.06.005 PMC8093649 33997176

[76] Godwin P Baird AM Heavey S Barr MP O’Byrne KJ Gately K. Targeting Nuclear Factor-Kappa B to Overcome Resistance to Chemotherapy. Front Oncol (2013) 3:120. doi: 10.3389/fonc.2013.00120 23720710 PMC3655421

[77] Gabrielson M Björklund MY Carlson J Shoshan M. Expression of Mitochondrial Regulators Pgc1α and TFAM as Putative Markers of Subtype and Chemoresistance in Epithelial Ovarian Carcinoma. PloS One (2014) 9:e107109–21. doi: 10.1371/JOURNAL.PONE.0107109 PMC4170973 25243473

[78] Yao Z Jones AWE Fassone E Sweeney MG Lebiedzinska M Suski JM. PGC-1β Mediates Adaptive Chemoresistance Associated With Mitochondrial DNA Mutations. Oncogene (2013) 32:2592–600. doi: 10.1038/ONC.2012.259 22777349

[79] Xie L Zhou T Xie Y Bode AM Cao Y. Mitochondria-Shaping Proteins and Chemotherapy. Front Oncol (2021) 11:769036/BIBTEX. doi: 10.3389/FONC.2021.769036/BIBTEX 34868997 PMC8637292

[80] Chang CR Blackstone C. Dynamic Regulation of Mitochondrial Fission Through Modification of the Dynamin-Related Protein Drp1. Ann N Y Acad Sci (2010) 1201:34–47. doi: 10.1111/J.1749-6632.2010.05629.X 20649536 PMC5585781

[81] Tailor D Hahm ER Kale RK Singh SV Singh RP. Sodium Butyrate Induces DRP1-Mediated Mitochondrial Fusion and Apoptosis in Human Colorectal Cancer Cells. Mitochondrion (2014) 16:55–64. doi: 10.1016/J.MITO.2013.10.004 24177748 PMC4004730

[82] Qian W Wang J Roginskaya V McDermott LA Edwards RP Stolz DB. Novel Combination of Mitochondrial Division Inhibitor 1 (Mdivi-1) and Platinum Agents Produces Synergistic Pro-Apoptotic Effect in Drug Resistant Tumor Cells. Oncotarget (2014) 5:4180–94. doi: 10.18632/ONCOTARGET.1944 PMC4147315 24952704

[83] Chiang YY Chen SL Hsiao YT Huang CH Lin TY Chiang IP. Nuclear Expression of Dynamin-Related Protein 1 in Lung Adenocarcinomas. Mod Pathol (2009) 22:1139–50. doi: 10.1038/MODPATHOL.2009.83 19525928

[84] Ferreira-da-Silva A Valacca C Rios E Pópulo H Soares P Sobrinho-Simões M. Mitochondrial Dynamics Protein Drp1 Is Overexpressed in Oncocytic Thyroid Tumors and Regulates Cancer Cell Migration. PloS One (2015) 10:e0122308–21. doi: 10.1371/journal.pone.0122308 PMC4379140 25822260

[85] Li W Melton DW. Cisplatin Regulates the MAPK Kinase Pathway to Induce Increased Expression of DNA Repair Gene ERCC1 and Increase Melanoma Chemoresistance. Oncogene (2011) 31:2412–22. doi: 10.1038/ONC.2011.426 21996734

[86] Zhang Y Cao S Zhuang C Chen J Chen X Sun H. ERCC1 Rs11615 Polymorphism and Chemosensitivity to Platinum Drugs in Patients With Ovarian Cancer: A Systematic Review and Meta-Analysis. J Ovarian Res (2021) 14:80–97. doi: 10.1186/s13048-021-00831-y 34148553 PMC8215742

[87] Han W Yin H Ma H Wang Y Kong D Fan Z. Curcumin Regulates ERCC1 Expression and Enhances Oxaliplatin Sensitivity in Resistant Colorectal Cancer Cells Through Its Effects on miR-409-3p. Evidence-Based Complement Altern Med (2020) 2020:1–16. doi: 10.1155/2020/8394574 PMC7519441 33014113

[88] Zhao H Yu X Ding Y Zhao J Wang G Wu X. MiR-770-5p Inhibits Cisplatin Chemoresistance in Human Ovarian Cancer by Targeting ERCC2. Oncotarget (2016) 7:53254–68. doi: 10.18632/ONCOTARGET.10736 PMC5288183 27449101

[89] Zhu X Zou S Zhou J Zhu H Zhang S Shang Z. Rev3l, the Catalytic Subunit of DNA Polymerase ζ, is Involved in the Progression and Chemoresistance of Esophageal Squamous Cell Carcinoma. Oncol Rep (2016) 35:1664–70. doi: 10.3892/or.2016.4549 26752104

[90] Wang W Sheng W Yu C Cao J Zhou J Wu J. REV3L Modulates Cisplatin Sensitivity of non-Small Cell Lung Cancer H1299 Cells. Oncol Rep (2015) 34:1460–8. doi: 10.3892/OR.2015.4121/HTML 26165320

[91] Shuhua W Chenbo S Yangyang L Xiangqian G Shuang H Tangyue L. Autophagy-Related Genes Raptor, Rictor, and Beclin1 Expression and Relationship With Multidrug Resistance in Colorectal Carcinoma. Hum Pathol (2015) 46:1752–9. doi: 10.1016/J.HUMPATH.2015.07.016 26363527

[92] Izdebska M Zielińska W Hałas-Wiśniewska M Grzanka A. Involvement of Actin in Autophagy and Autophagy-Dependent Multidrug Resistance in Cancer. Cancers (Basel) (2019) 11:1209–20. doi: 10.3390/cancers11081209 PMC6721626 31434275

[93] Rikiishi H. Novel Insights Into the Interplay Between Apoptosis and Autophagy. Int J Cell Biol (2012) 2012:1–14. doi: 10.1155/2012/317645 PMC3312193 22496691

[94] Yang L Yu Y Kang R Yang M Xie M Wang Z. Up-Regulated Autophagy by Endogenous High Mobility Group Box-1 Promotes Chemoresistance in Leukemia Cells. Leuk Lymphoma (2012) 53:315–22. doi: 10.3109/10428194.2011.616962 21864037

[95] Wang L Zhang H Sun M Yin Z Qian J. High Mobility Group Box 1-Mediated Autophagy Promotes Neuroblastoma Cell Chemoresistance. Oncol Rep (2015) 34:2969–76. doi: 10.3892/OR.2015.4278/HTML 26397184

[96] Lamouille S Xu J Derynck R. Molecular Mechanisms of Epithelial-Mesenchymal Transition. Nat Rev Mol Cell Biol (2014) 15:178–96. doi: 10.1038/NRM3758 PMC4240281 24556840

[97] Fischer KR Durrans A Lee S Sheng J Li F Wong STC. Epithelial-To-Mesenchymal Transition is Not Required for Lung Metastasis But Contributes to Chemoresistance. Nature (2015) 527:472–6. doi: 10.1038/NATURE15748 PMC4662610 26560033

[98] Li J Liu H Yu J Yu H. Chemoresistance to Doxorubicin Induces Epithelial-Mesenchymal Transition *via* Upregulation of Transforming Growth Factor β Signaling in HCT116 Colon Cancer Cells. Mol Med Rep (2015) 12:192–8. doi: 10.3892/MMR.2015.3356 PMC4438913 25684678

[99] Wang R Li Y Hou Y Yang Q Chen S Wang X. The PDGF-D/miR-106a/Twist1 Pathway Orchestrates Epithelial-Mesenchymal Transition in Gemcitabine Resistance Hepatoma Cells. Oncotarget (2015) 6:7000–10. doi: 10.18632/oncotarget.3193 PMC4466665 25760076

[100] Ma J Zeng S Zhang Y Deng G Qu Y Guo C. BMP4 Promotes Oxaliplatin Resistance by an Induction of Epithelial-Mesenchymal Transition *via* MEK1/ERK/ELK1 Signaling in Hepatocellular Carcinoma. Cancer Lett (2017) 411:117–29. doi: 10.1016/j.canlet.2017.09.041 28987388

[101] Zhang W Feng M Zheng G Chen Y Wang X Pen B. Chemoresistance to 5-Fluorouracil Induces Epithelial-Mesenchymal Transition *via* Up-Regulation of Snail in MCF7 Human Breast Cancer Cells. Biochem Biophys Res Commun (2012) 417:679–85. doi: 10.1016/j.bbrc.2011.11.142 22166209

[102] Reya T Morrison SJ Clarke MF Weissman IL. Stem Cells, Cancer, and Cancer Stem Cells. Nature (2001) 414:105–11. doi: 10.1038/35102167 11689955

[103] Batlle E Clevers H. Cancer Stem Cells Revisited. Nat Med (2017) 23:1124–34. doi: 10.1038/NM.4409 28985214

[104] Hirschmann-Jax C Foster AE Wulf GG Nuchtern JG Jax TW Gobel U. A Distinct “Side Population” of Cells With High Drug Efflux Capacity in Human Tumor Cells. Proc Natl Acad Sci (2004) 101:14228–33. doi: 10.1073/pnas.0400067101 PMC521140 15381773

[105] Zhou S Schuetz JD Bunting KD Colapietro AM Sampath J Morris JJ. The ABC Transporter Bcrp1/ABCG2 is Expressed in a Wide Variety of Stem Cells and is a Molecular Determinant of the Side-Population Phenotype. Nat Med (2001) 7:1028–34. doi: 10.1038/NM0901-1028 11533706

[106] Qiu L Li H Fu S Chen X Lu L. Surface Markers of Liver Cancer Stem Cells and Innovative Targeted-Therapy Strategies for HCC (Review). Oncol Lett (2017) 15:2039–48. doi: 10.3892/ol.2017.7568 PMC5776936 29434903

[107] Munro MJ Wickremesekera SK Peng L Tan ST Itinteang T. Cancer Stem Cells in Colorectal Cancer: A Review. J Clin Pathol (2018) 71:110–6. doi: 10.1136/JCLINPATH-2017-204739 28942428

[108] Zhang X Powell K Li L. Breast Cancer Stem Cells: Biomarkers, Identification and Isolation Methods, Regulating Mechanisms, Cellular Origin, and Beyond. Cancers (Basel) (2020) 12:1–28. doi: 10.3390/CANCERS12123765 PMC7765014 33327542

[109] Capulli M Hristova D Valbret Z Carys K Arjan R Maurizi A. Notch2 Pathway Mediates Breast Cancer Cellular Dormancy and Mobilisation in Bone and Contributes to Haematopoietic Stem Cell Mimicry. Br J Cancer (2019) 121:157–71. doi: 10.1038/s41416-019-0501-y PMC6738045 31239543

[110] Todaro M Gaggianesi M Catalano V Benfante A Iovino F Biffoni M. CD44v6 is a Marker of Constitutive and Reprogrammed Cancer Stem Cells Driving Colon Cancer Metastasis. Cell Stem Cell (2014) 14:342–56. doi: 10.1016/J.STEM.2014.01.009 24607406

[111] Gao Y Foster R Yang X Feng Y Shen JK Mankin HJ. Up-Regulation of CD44 in the Development of Metastasis, Recurrence and Drug Resistance of Ovarian Cancer. Oncotarget (2015) 6:9313–26. doi: 10.18632/ONCOTARGET.3220 PMC4496219 25823654

[112] Curley MD Therrien VA Cummings CL Sergent PA Koulouris CR Friel AM. CD133 Expression Defines a Tumor Initiating Cell Population in Primary Human Ovarian Cancer. Stem Cells (2009) 27:2875–83. doi: 10.1002/STEM.236 19816957

[113] Mueller MM Fusenig NE. Friends or Foes - Bipolar Effects of the Tumour Stroma in Cancer. Nat Rev Cancer (2004) 4:839–49. doi: 10.1038/NRC1477 15516957

[114] Zhao L Chen J Pang Y Fu K Shang Q Wu H. Fibroblast Activation Protein-Based Theranostics in Cancer Research: A State-of-the-Art Review. Theranostics (2022) 12:1557–69. doi: 10.7150/thno.69475 PMC8825585 35198057

[115] Loeffler M Krüger JA Niethammer AG Reisfeld RA. Targeting Tumor-Associated Fibroblasts Improves Cancer Chemotherapy by Increasing Intratumoral Drug Uptake. J Clin Invest (2006) 116:1955–62. doi: 10.1172/JCI26532 PMC1481657 16794736

[116] Ruocco MR Avagliano A Granato G Imparato V Masone S Masullo M. Involvement of Breast Cancer-Associated Fibroblasts in Tumor Development, Therapy Resistance and Evaluation of Potential Therapeutic Strategies. Curr Med Chem (2018) 25:3414–34. doi: 10.2174/0929867325666180309120746 29521203

[117] Qiao Y Zhang C Li A Wang D Luo Z Ping Y. IL6 Derived From Cancer-Associated Fibroblasts Promotes Chemoresistance *via* CXCR7 in Esophageal Squamous Cell Carcinoma. Oncogene (2018) 37:873–83. doi: 10.1038/onc.2017.387 29059160

[118] Zhai J Shen J Xie G Wu J He M Gao L. Cancer-Associated Fibroblasts-Derived IL-8 Mediates Resistance to Cisplatin in Human Gastric Cancer. Cancer Lett (2019) 454:37–43. doi: 10.1016/j.canlet.2019.04.002 30978440

[119] Lotti F Jarrar AM Pai RK Hitomi M Lathia J Mace A. Chemotherapy Activates Cancer-Associated Fibroblasts to Maintain Colorectal Cancer-Initiating Cells by IL-17a. J Exp Med (2013) 210:2851–72. doi: 10.1084/jem.20131195 PMC3865474 24323355

[120] Valenti G Quinn HM Heynen GJJE Lan L Holland JD Vogel R. Cancer Stem Cells Regulate Cancer-Associated Fibroblasts *via* Activation of Hedgehog Signaling in Mammary Gland Tumors. Cancer Res (2017) 77:2134–47. doi: 10.1158/0008-5472.CAN-15-3490 28202523

[121] Al Tameemi W Dale TP Al-Jumaily RMK Forsyth NR. Hypoxia-Modified Cancer Cell Metabolism. Front Cell Dev Biol (2019) 7:4. doi: 10.3389/fcell.2019.00004 30761299 PMC6362613

[122] Apicella M Giannoni E Fiore S Ferrari KJ Fernández-Pérez D Isella C. Increased Lactate Secretion by Cancer Cells Sustains Non-Cell-Autonomous Adaptive Resistance to MET and EGFR Targeted Therapies. Cell Metab (2018) 28:848–65.e6. doi: 10.1016/j.cmet.2018.08.006 30174307

[123] Kuen J Darowski D Kluge T Majety M. Pancreatic Cancer Cell/Fibroblast Co-Culture Induces M2 Like Macrophages That Influence Therapeutic Response in a 3D Model. PloS One (2017) 12:e0182039–54. doi: 10.1371/journal.pone.0182039 PMC5531481 28750018

[124] Cohen N Shani O Raz Y Sharon Y Hoffman D Abramovitz L. Fibroblasts Drive an Immunosuppressive and Growth-Promoting Microenvironment in Breast Cancer *via* Secretion of Chitinase 3-Like 1. Oncogene (2017) 36:4457–68. doi: 10.1038/onc.2017.65 PMC5507301 28368410

[125] Li C Xu X. Biological Functions and Clinical Applications of Exosomal non-Coding RNAs in Hepatocellular Carcinoma. Cell Mol Life Sci (2019) 76:4203–19. doi: 10.1007/s00018-019-03215-0 PMC11105530 31300868

[126] Safaei R Larson BJ Cheng TC Gibson MA Otani S Naerdemann W. Abnormal Lysosomal Trafficking and Enhanced Exosomal Export of Cisplatin in Drug-Resistant Human Ovarian Carcinoma Cells. Mol Cancer Ther (2005) 4:1595–604. doi: 10.1158/1535-7163.MCT-05-0102 16227410

[127] Muralidharan-Chari V Kohan HG Asimakopoulos AG Sudha T Sell S Kannan K. Microvesicle Removal of Anticancer Drugs Contributes to Drug Resistance in Human Pancreatic Cancer Cells. Oncotarget (2016) 7:50365–79. doi: 10.18632/oncotarget.10395 PMC5226588 27391262

[128] Efferth T Volm M. Multiple Resistance to Carcinogens and Xenobiotics: P-Glycoproteins as Universal Detoxifiers. Arch Toxicol (2017) 91:2515–38. doi: 10.1007/s00204-017-1938-5 28175954

[129] Gong J Luk F Jaiswal R George AM Grau GER Bebawy M. Microparticle Drug Sequestration Provides a Parallel Pathway in the Acquisition of Cancer Drug Resistance. Eur J Pharmacol (2013) 721:116–25. doi: 10.1016/J.EJPHAR.2013.09.044 24095666

[130] Ifergan I Scheffer GL Assaraf YG. Novel Extracellular Vesicles Mediate an ABCG2-Dependent Anticancer Drug Sequestration and Resistance. Cancer Res (2005) 65:10952–8. doi: 10.1158/0008-5472.CAN-05-2021 16322243

[131] Goler-Baron V Assaraf YG. Overcoming Multidrug Resistance *via* Photodestruction of ABCG2-Rich Extracellular Vesicles Sequestering Photosensitive Chemotherapeutics. PLoS One (2012) 7:e35487. doi: 10.1371/journal.pone.0035487 22530032 PMC3329466

[132] Cappariello A Rucci N. Tumour-Derived Extracellular Vesicles (EVs): A Dangerous “Message in a Bottle” for Bone. Int J Mol Sci (2019) 20:4805–21. doi: 10.3390/ijms20194805 PMC6802008 31569680

[133] Muraca M Cappariello A. The Role of Extracellular Vesicles (EVs) in the Epigenetic Regulation of Bone Metabolism and Osteoporosis. Int J Mol Sci (2020) 21:8682–703. doi: 10.3390/ijms21228682 PMC7698531 33213099

[134] Schmidt LH Spieker T Koschmieder S Humberg J Jungen D Bulk E. The Long Noncoding MALAT-1 RNA Indicates a Poor Prognosis in non-Small Cell Lung Cancer and Induces Migration and Tumor Growth. J Thorac Oncol (2011) 6:1984–92. doi: 10.1097/JTO.0B013E3182307EAC 22088988

[135] Lin R Maeda S Liu C Karin M Edgington TS. A Large Noncoding RNA is a Marker for Murine Hepatocellular Carcinomas and a Spectrum of Human Carcinomas. Oncogene (2007) 26:851–8. doi: 10.1038/SJ.ONC.1209846 16878148

[136] Gezer U Özgür E Cetinkaya M Isin M Dalay N. Long non-Coding RNAs With Low Expression Levels in Cells are Enriched in Secreted Exosomes. Cell Biol Int (2014) 38:1076–9. doi: 10.1002/CBIN.10301 24798520

[137] Kogure T Yan IK Lin WL Patel T. Extracellular Vesicle-Mediated Transfer of a Novel Long Noncoding RNA TUC339: A Mechanism of Intercellular Signaling in Human Hepatocellular Cancer. Genes Cancer (2013) 4:261–72. doi: 10.1177/1947601913499020 PMC3807642 24167654

[138] Braconi C Valeri N Kogure T Gasparini P Huang N Nuovo GJ. Expression and Functional Role of a Transcribed Noncoding RNA With an Ultraconserved Element in Hepatocellular Carcinoma. Proc Natl Acad Sci U S A (2011) 108:786–91. doi: 10.1073/PNAS.1011098108 PMC3021052 21187392

[139] Yoshida A Fujiwara T Uotani K Morita T Kiyono M Yokoo S. Clinical and Functional Significance of Intracellular and Extracellular microRNA-25-3p in Osteosarcoma. Acta Med Okayama (2018) 72:165–74. doi: 10.18926/AMO/55857 29674765

[140] Pan Y Lin Y Mi C. Cisplatin-Resistant Osteosarcoma Cell-Derived Exosomes Confer Cisplatin Resistance to Recipient Cells in an Exosomal Circ_103801-Dependent Manner. Cell Biol Int (2021) 45:858–68. doi: 10.1002/CBIN.11532 33325136

[141] Wei Y Lai X Yu S Chen S Ma Y Zhang Y. Exosomal miR-221/222 Enhances Tamoxifen Resistance in Recipient ER-Positive Breast Cancer Cells. Breast Cancer Res Treat (2014) 147:423–31. doi: 10.1007/s10549-014-3037-0 25007959

[142] Oun R Moussa YE Wheate NJ. The Side Effects of Platinum-Based Chemotherapy Drugs: A Review for Chemists. Dalt Trans (2018) 47:6645–53. doi: 10.1039/C8DT00838H 29632935

[143] Weinman MA Ramsey SA Leeper HJ Brady JV Schlueter A Stanisheuski S. Exosomal Proteomic Signatures Correlate With Drug Resistance and Carboplatin Treatment Outcome in a Spontaneous Model of Canine Osteosarcoma. Cancer Cell Int (2021) 21:245–63. doi: 10.1186/s12935-021-01943-7 PMC8088716 33933069

[144] Iba K Durkin ME Johnsen L Hunziker E Damgaard-Pedersen K Zhang H. Mice With a Targeted Deletion of the Tetranectin Gene Exhibit a Spinal Deformity. Mol Cell Biol (2001) 21:7817–25. doi: 10.1128/MCB.21.22.7817-7825.2001 PMC99951 11604516

[145] Ren J Ding L Zhang D Shi G Xu Q Shen S. Carcinoma-Associated Fibroblasts Promote the Stemness and Chemoresistance of Colorectal Cancer by Transferring Exosomal lncRNA H19. Theranostics (2018) 8:3932–48. doi: 10.7150/thno.25541 PMC6071523 30083271

[146] Lin D Zhang H Liu R Deng T Ning T Bai M. iRGD-Modified Exosomes Effectively Deliver CPT1A siRNA to Colon Cancer Cells, Reversing Oxaliplatin Resistance by Regulating Fatty Acid Oxidation. Mol Oncol (2021) 15:3430–46. doi: 10.1002/1878-0261.13052 PMC8637580 34213835

[147] Hui B Lu C Wang J Xu Y Yang Y Ji H. Engineered Exosomes for Co-Delivery of PGM5-AS1 and Oxaliplatin to Reverse Drug Resistance in Colon Cancer. J Cell Physiol (2022) 237:911–33. doi: 10.1002/jcp.30566 34463962

[148] Xiao Z Liu Y Li Q Liu Q Liu Y Luo Y. EVs Delivery of miR-1915-3p Improves the Chemotherapeutic Efficacy of Oxaliplatin in Colorectal Cancer. Cancer Chemother Pharmacol (2021) 88:1021–31. doi: 10.1007/s00280-021-04348-5 34599680

[149] Hon KW Ab-Mutalib NS Abdullah NMA Jamal R Abu N. Extracellular Vesicle-Derived Circular RNAs Confers Chemoresistance in Colorectal Cancer. Sci Rep (2019) 9:16497. doi: 10.1038/s41598-019-53063-y 31712601 PMC6848089

[150] Boelens MC Wu TJ Nabet BY Xu B Qiu Y Yoon T. Exosome Transfer From Stromal to Breast Cancer Cells Regulates Therapy Resistance Pathways. Cell (2014) 159:499–513. doi: 10.1016/j.cell.2014.09.051 25417103 PMC4283810

[151] Longley DB Harkin DP Johnston PG. 5-Fluorouracil: Mechanisms of Action and Clinical Strategies. Nat Rev Cancer 2003 35 (2003) 3:330–8. doi: 10.1038/nrc1074 12724731

[152] Zhao K Cheng X Ye Z Li Y Peng W Wu Y. Exosome-Mediated Transfer of Circ_0000338 Enhances 5-Fluorouracil Resistance in Colorectal Cancer Through Regulating MicroRNA 217 (miR-217) and miR-485-3p. Mol Cell Biol (2021) 41:e00517–20. doi: 10.1128/MCB.00517-20 PMC8088270 33722958

[153] Ji R Zhang B Zhang X Xue J Yuan X Yan Y. Exosomes Derived From Human Mesenchymal Stem Cells Confer Drug Resistance in Gastric Cancer. Cell Cycle (2015) 14:2473–83. doi: 10.1080/15384101.2015.1005530 PMC4613597 26091251

[154] Hu JL Wang W Lan XL Zeng ZC Liang YS Yan YR. CAFs Secreted Exosomes Promote Metastasis and Chemotherapy Resistance by Enhancing Cell Stemness and Epithelial-Mesenchymal Transition in Colorectal Cancer. Mol Cancer (2019) 18:91–105. doi: 10.1186/s12943-019-1019-x 31064356 PMC6503554

[155] Zheng X Ma N Wang X Hu J Ma X Wang J. Exosomes Derived From 5-Fluorouracil-Resistant Colon Cancer Cells are Enriched in GDF15 and can Promote Angiogenesis. J Cancer (2020) 11:7116–26. doi: 10.7150/jca.49224 PMC7646166 33193874

[156] Fu X Liu M Qu S Ma J Zhang Y Shi T. Exosomal microRNA-32-5p Induces Multidrug Resistance in Hepatocellular Carcinoma *via* the PI3K/Akt Pathway. J Exp Clin Cancer Res (2018) 37:52–68. doi: 10.1186/s13046-018-0677-7 29530052 PMC5846230

[157] Tacar O Sriamornsak P Dass CR. Doxorubicin: An Update on Anticancer Molecular Action, Toxicity and Novel Drug Delivery Systems. J Pharm Pharmacol (2012) 65:157–70. doi: 10.1111/J.2042-7158.2012.01567.X 23278683

[158] Torreggiani E Roncuzzi L Perut F Zini N Baldini N. Multimodal Transfer of MDR by Exosomes in Human Osteosarcoma. Int J Oncol (2016) 49:189–96. doi: 10.3892/ijo.2016.3509 27176642

[159] Wang W Zou L Zhou D Zhou Z Tang F Xu Z. Overexpression of Ubiquitin Carboxyl Terminal Hydrolase-L1 Enhances Multidrug Resistance and Invasion/Metastasis in Breast Cancer by Activating the MAPK/Erk Signaling Pathway. Mol Carcinog (2016) 55:1329–42. doi: 10.1002/mc.22376 26293643

[160] Yang SJ Wang DD Jian L Xu HZ Shen HY Chen X. Predictive Role of GSTP1-Containing Exosomes in Chemotherapy-Resistant Breast Cancer. Gene (2017) 623:5–14. doi: 10.1016/J.GENE.2017.04.031 28438694

[161] Takahashi K Yan IK Wood J Haga H Patel T. Involvement of Extracellular Vesicle Long Noncoding RNA (Linc-VLDLR) in Tumor Cell Responses to Chemotherapy. Mol Cancer Res (2014) 12:1377–87. doi: 10.1158/1541-7786.MCR-13-0636 PMC4201956 24874432

[162] Weaver BA. How Taxol/paclitaxel Kills Cancer Cells. Mol Biol Cell (2014) 25:2677–81. doi: 10.1091/mbc.E14-04-0916 PMC4161504 25213191

[163] LV M-M Zhu XY Chen WX Zhong SL Hu Q Ma TF. Exosomes Mediate Drug Resistance Transfer in MCF-7 Breast Cancer Cells and a Probable Mechanism is Delivery of P-Glycoprotein. Tumor Biol (2014) 35:10773–9. doi: 10.1007/s13277-014-2377-z 25077924

[164] Kreger B Johansen E Cerione R Antonyak M. The Enrichment of Survivin in Exosomes From Breast Cancer Cells Treated With Paclitaxel Promotes Cell Survival and Chemoresistance. Cancers (Basel) (2016) 8:111–27. doi: 10.3390/cancers8120111 PMC5187509 27941677

[165] Shan G Gu J Zhou D Li L Cheng W Wang Y. Cancer-Associated Fibroblast-Secreted Exosomal miR-423-5p Promotes Chemotherapy Resistance in Prostate Cancer by Targeting GREM2 Through the TGF-β Signaling Pathway. Exp Mol Med (2020) 52:1809–22. doi: 10.1038/s12276-020-0431-z PMC8080786 33144675

[166] Zhang S Zhang Y Qu J Che X Fan Y Hou K. Exosomes Promote Cetuximab Resistance *via* the PTEN/Akt Pathway in Colon Cancer Cells. Braz J Med Biol Res (2018) 51:e6472–87. doi: 10.1590/1414-431x20176472 PMC5685060 29160412

[167] Ciravolo V Huber V Ghedini GC Venturelli E Bianchi F Campiglio M. Potential Role of HER2-Overexpressing Exosomes in Countering Trastuzumab-Based Therapy. J Cell Physiol (2012) 227:658–67. doi: 10.1002/jcp.22773 21465472

[168] Maury S Chevret S Thomas X Heim D Leguay T Huguet F. Rituximab in B-Lineage Adult Acute Lymphoblastic Leukemia. N Engl J Med (2016) 375:1044–53. doi: 10.1056/NEJMoa1605085 27626518

[169] Aung T Chapuy B Vogel D Wenzel D Oppermann M Lahmann M. Exosomal Evasion of Humoral Immunotherapy in Aggressive B-Cell Lymphoma Modulated by ATP-Binding Cassette Transporter A3. Proc Natl Acad Sci U S A (2011) 108:15336–41. doi: 10.1073/pnas.1102855108 PMC3174603 21873242

[170] Lubin JA Zhang RR Kuo JS. Extracellular Vesicles Containing PD-L1 Contribute to Immune Evasion in Glioblastoma. Neurosurgery (2018) 83:E98–E100. doi: 10.1093/neuros/nyy295 30125027

[171] Chen G Huang AC Zhang W Zhang G Wu M Xu W. Exosomal PD-L1 Contributes to Immunosuppression and is Associated With Anti-PD-1 Response. Nature (2018) 560:382–6. doi: 10.1038/s41586-018-0392-8 PMC6095740 30089911

[172] Ma J Zhang Y Tang K Zhang H Yin X Li Y. Reversing Drug Resistance of Soft Tumor-Repopulating Cells by Tumor Cell-Derived Chemotherapeutic Microparticles. Cell Res (2016) 26:713–27. doi: 10.1038/cr.2016.53 PMC4897183 27167569

[173] Saari H Lázaro-Ibáñez E Viitala T Vuorimaa-Laukkanen E Siljander P Yliperttula M. Microvesicle- and Exosome-Mediated Drug Delivery Enhances the Cytotoxicity of Paclitaxel in Autologous Prostate Cancer Cells. J Control Release (2015) 220:727–37. doi: 10.1016/j.jconrel.2015.09.031 26390807

[174] Kim MS Haney MJ Zhao Y Yuan D Deygen I Klyachko NL. Engineering Macrophage-Derived Exosomes for Targeted Paclitaxel Delivery to Pulmonary Metastases: *In Vitro* and *In Vivo* Evaluations. Nanomed Nanotechnol Biol Med (2018) 14:195–204. doi: 10.1016/j.nano.2017.09.011 28982587

[175] Lee H Park H Noh GJ Lee ES. pH-Responsive Hyaluronate-Anchored Extracellular Vesicles to Promote Tumor-Targeted Drug Delivery. Carbohydr Polym (2018) 202:323–33. doi: 10.1016/j.carbpol.2018.08.141 30287007

